# Strategic emerging industry layout based on analytic hierarchy process and fuzzy comprehensive evaluation: A case study of Sichuan province

**DOI:** 10.1371/journal.pone.0264578

**Published:** 2022-03-04

**Authors:** Kai He, Nan Zhu

**Affiliations:** 1 School of Statistics, Southwestern University of Finance and Economics, Chengdu, China; 2 Western Business School, Southwestern University of Finance and Economics, Chengdu, China; Gonbad Kavous University, ISLAMIC REPUBLIC OF IRAN

## Abstract

In the 12th Five-Year Plan period, Sichuan province has selected strategic emerging industries such as new-generation information technology, new energy, high-end equipment manufacturing, new materials, biology, energy conservation and environmental protection. However, its strategic emerging industries are still in the stage of cultivation and development, and there are some problems such as low industrial agglomeration and obvious industrial restructuring. In essence, this is because the focus of the development of strategic emerging industries is not clear enough, and the location advantages are not fully utilized to select the most suitable strategic emerging industries for the development of the region. This paper constructs a regional strategic emerging industries selection decision model, applies ARCGIS to study the spatial distribution of strategic emerging industries in Sichuan province, and uses fuzzy comprehensive evaluation method (FCEM) and analytic hierarchy process (AHP) to solve the priority of developing strategic emerging industries. Conclusions: Firstly, Sichuan province should give priority to the development of new generation information technology industry and new energy vehicle industry, then high-end equipment manufacturing industry, energy-saving and environmental protection industry and new energy industry, and finally biological industry and new material industry. The larger the coefficient of influence is, the higher the comprehensive score is, while the larger the coefficient of sensitivity is, the lower the comprehensive score is. Secondly, the number of enterprises in the new-generation information technology industry and the new energy vehicle industry is still not dominant in Sichuan province. Finally, the study found that the current development of strategic emerging industries in Sichuan province is extremely unbalanced in various regions, and the phenomenon of competition and reconstruction is obvious.

## Introduction

Strategic emerging industries are based on major technological breakthroughs and major development needs, and represent the direction of a new round of scientific and technological revolution and industrial transformation. Accelerating the development of strategic emerging industries is an important measure to implement the innovation-driven development strategy and advance supply-side structural reform. Since the outbreak of the international financial crisis, major countries in the world have sought solutions to the crisis and accelerated the planning and distribution of strategic emerging industries to release more space for economic development and foster new economic growth points. Specifically, the U.S. has formulated the "American Innovation Strategy" and the "Retooling American Manufacturing Framework" to support the development of clean energy, bioengineering, nanotechnology, and smart grid. The European Union has put forward the Strategic Plan on Energy Technology and the European Economic Recovery Plan, focusing on supporting and developing materials and new product technology, nano engineering, and agricultural bioengineering. In the UK, there are "Shaping Britain’s Future" and "Digital Britain", which pay more attention to low-carbon economy, life sciences, digital economy, cash manufacturing and financial services. Not to be outdone, Japan has put forward a low-carbon Social Action Plan and a New Growth Strategy for a Glorious Japan, aiming to make breakthroughs in environment-friendly vehicles, electric vehicles, and the application of information technology for solar power generation. Adhering to the basic principle of the development of strategic emerging industries, China has established seven strategic emerging industries, including energy conservation and environmental protection, new generation information technology, biology, high-end equipment manufacturing, new energy, new materials, and new energy vehicles, according to the meaning and characteristics of strategic emerging industries and in combination with the experience of foreign emerging industry selection.

During the 12th Five-Year Plan period, Sichuan province selected strategic emerging industries such as new-generation information technology, new energy, high-end equipment manufacturing, new materials, biology and energy conservation and environmental protection. In 2015, the total output value of Sichuan’s strategic emerging industries reached 567.15 billion yuan, 2.5 times that of 2010, accounting for 13.9% of the province’s total output value of industries above designated size, 4.5 percentage points higher than that of the same period in 2010. It should be said that the strength of strategic emerging industries in Sichuan province has been significantly enhanced, which has played a very important role in stabilizing growth, adjusting structure, promoting transformation, and benefiting people’s livelihood. But at the same time, during the 13th Five-Year Plan period, Sichuan province shoulders the glorious mission of revitalizing the west, with arduous tasks and heavy responsibilities. At present, Sichuan Province has created a good institutional and policy environment for the development of strategic emerging industries, but its strategic emerging industries are still in the cultivation stage, and there are many problems, such as incomplete industrial chain, weak industrial support ability, weak industrial independent innovation ability, low industrial added value, weak market cultivation and industrial competitiveness. These problems are essentially due to the irrationality of the spatial layout of strategic emerging industries. When [1] studied the layout of strategic emerging industries by taking Beijing as an example, he believed that the problems in the spatial layout of China’s strategic emerging industries were mainly reflected in the following aspects: Firstly, the agglomeration degree of industrial spatial distribution was not high; Secondly, inter-regional industrial restructuring phenomenon (It refers to the phenomenon of the same industrial function, similar structure and repeated construction in various regions) is more obvious; Thirdly, the industrial development environment is out of sync with industrial planning. In addition, we believe that the development of strategic emerging industries in various regions still has the problems of seeking quick success and instant benefits, over-relying on scale expansion, and not closely combining the regional advantages in the choice of industries.

Therefore, it is necessary to conduct a more in-depth analysis of the spatial layout of strategic emerging industries, study which factors will affect their spatial layout, and build a relatively complete decision-making system for the layout of related industries, and provide necessary guidance for the selection of regional strategic emerging industries in practice.

The following parts of this paper are arranged as follows: Section 2 makes a literature review related to this research; Section 3 describes the materials and methods used in the study, and the results and discussion sections are carried out in sections 4 and 5 respectively; Section 6 draws the conclusion of this paper.

## Literature review

Strategic emerging industries were first proposed in The State Council Strategic Emerging Industries Development Symposium in 2009, and then in 2010, The State Council’s Decision on Accelerating the Cultivation and Development of Strategic Emerging Industries (hereinafter referred to as the Decision) was issued. It has formally defined strategic emerging industries as "industries with intensive knowledge and technology, low consumption of material resources, great growth potential and good comprehensive returns based on major technological breakthroughs and major development needs, which play a leading role in overall economic and social development and long-term development."

There is still not unified standard for the definition of strategic emerging industry in China’s theoretical circle. [2] believes that strategic emerging industries are linked with national economic and social development and industrial structure upgrading, and have the characteristics of overall, long-term, guiding, and dynamic. This shows that strategic emerging industries play a vital role in the long-term development of the country and the overall direction of the national economy. [3] understood the connotation of strategic emerging industries from three aspects: "breakthrough technological innovation", "strategic" and "serving national strategic objectives". Based on international and domestic typical cases, [4] analyzes the advantages and disadvantages of Zhoushan Marine strategic emerging industries in China, determines the selection criteria, studies the development direction and methods of Zhoushan Marine strategic emerging industries, and builds a hierarchical cultivation system of strategic emerging industries. [5] uses the fuzzy optimization theory to select the regional central industry as the regional strategic emerging industry, and tries to optimize the weight calculation of multi-level fuzzy comprehensive evaluation to obtain more accurate results. [6] adopts SWOT analysis to study the talents of strategic emerging industries in Henan Province. [7] used Logistic model to characterize the life cycle of strategic emerging industry clusters. A scale-free evolution model based on the growth rate and grey number optimization of strategic emerging industry cluster is introduced to analyze the stable state of strategic emerging industry cluster network. Most scholars agree that vigorously developing strategic emerging industries can improve technological innovation ability and regional competitiveness. The strategic emerging industry refers to the industry with high technology content, low resource consumption, great growth potential and good comprehensive benefits.

The industrial layout is affected by many factors such as historical conditions, technological knowledge, and environmental factors. Industrial layout, also called industrial distribution and industrial allocation, refers to the distribution and organization of industries in a certain regional space. Simply put, the problem of industrial layout is "where to produce". [8] pointed out that the spatial layout of strategic emerging industries is a process in which the spatial "fragmented" distribution of the industrial value chain, the positioning of core value activities and functions and collaborative development are carried out based on the characteristics of strategic emerging industries and regional advantages. This paper holds that industrial spatial layout is an activity that rationally plans people’s productive activities in geographical space through points, lines, and planes to obtain better benefits.

Strategic emerging industry, from its literal point of view, is strategic industry and emerging industry. Outside China, the term "Emerging Industries" and "Strategic Industry" is commonly used, but there is no specific "Strategic Emerging Industry". [9] calculated the distribution of patents through the location entropy method, and the study showed that patents, technology licensing and product diversity all affect the regional distribution of patents. [10] used social network analysis to account for the spatial diffusion process of technological innovation. [11] discussed the extent to which the spatial distribution of economic promotes innovation. Using patent data of India from 1999 to 2007, it was concluded that R&D expenditure, diversity of industrial distribution and human capital endowment had a crucial impact on innovation through quantitative analysis. [12] discussed six regional development institutions in Canada, adopted the perspective of organizational learning to explain the shift of technological paradigm, and combined the traditional regional economic research model with the multi-level governance of government intervention to explain the causes of regional spatial change and development in Canada. [13] analyzed the spatial distribution characteristics and preferences of green industries in South Korea by using panel data from 2006 to 2012, and pointed out that such emerging industries are more likely to gather in areas where traditional manufacturing industries exist. [14] studied the spatial distribution pattern of traditional industry and innovative industry in Indonesia, believing that innovative industry is more dependent on human resources and financial diversification and tends to be distributed in large cities. This paper mainly studies the layout of strategic emerging industries, and it is a decision-making problem to choose which industries to pay attention to. Research on strategic emerging industries also includes financing efficiency, such as [15–17], technological innovation efficiency research, such as [18–21].

In China, most of the research on strategic emerging industries focus on the qualitative discussion of their connotation, characteristics, existing problems and policy suggestions, and the quantitative calculation of the degree of agglomeration, regional distribution, and spatial distribution of strategic emerging industries. According to the classification of strategic emerging industries (Trial) compiled by the National Bureau of statistics, [22] selected 18 categories of strategic emerging industries, and then analyzed the agglomeration degree and evolution trend of China’s strategic emerging industries by using the geographical concentration index method. The author also investigated the regional distribution characteristics and put forward the spatial layout plan of "one belt, two regions and multiple points" of China’s strategic emerging industries. [23] studied the efficiency of China’s strategic emerging industries by using data envelopment analysis, analyzed the spatial relevance of strategic emerging industries, and carried out spatial econometric statistical modeling for manufacturing and service industries respectively according to the relevant results. [24] analyzed the spatial differences of China’s regional circulation industry by using Theil coefficient and the dynamic evolution of the industrial center of gravity and the population center of gravity, and analyzed the spatial correlation of China’s regional circulation industry by using Moran’s I index and Moran scatter plot. Finally, the influencing factors of regional circulation industry development are analyzed by using traditional panel data and spatial panel data. [25] used a spatial econometric model to calculate the influencing factors of innovation clusters in 31 provinces, autonomous regions, and municipalities directly under the Central Government in China.

Fuzzy logic was proposed by [26] to deal with uncertainties of non-quantitative or statistical nature. Fuzzy concept is widely used in uncertainty models in real life. This method has been applied to management, economy, policy, engineering, and other fields. [27] used FCEM to evaluate venture capitalists’ projects and AHP to determine the weight of evaluation factors. [28] applied entropy FCEM to the evaluation of power system black start scheme, which is very important for the rapid recovery of the power system. [29] also utilized FCEM to evaluate the performance of state-owned enterprises. The Delphi method and entropy weight method are used to determine the weight of each variable and evaluate the performance of four state-owned enterprises in the field of real estate and project management. FCEM is an approach based on fuzzy logic that can model the subjective speech responses of different experts ([30]). FCEM is one of the multi-stage evaluation techniques used to organize, analyze, and evaluate complex decisions. It is especially used in group decision-making to obtain the decision most suitable for understanding potential problems. FCEM uses fuzzy logic to systematically evaluate undefined real-world systems. FCEM consists of the weight of the evaluation factor subset, the comment set and the members of the factor set.

Commonly used multi-criterion decision-making (MCDM) methods include Best Worst Method (BWM), Complex Proportional Assessment (COPRAS), Weighted Aggregates Sum Product Assessment (WASPAS), Simultaneous Evaluation of Criteria and Alternatives (SECA), Method based on the Removal Effects of Criteria (MEREC), Evaluation Based on Distance from Average Solution (EDAS), Technique for Order of Preference by Similarity to Ideal Solution (TOPSIS) and AHP. Best worst method (BWM) is a multi-criterion decision-making method, which uses two comparison vectors to determine the weight. The decision maker determines the best and worst standards, then compares the best standard with other standards and compares other standards with the worst standard. Then, the nonlinear min max model is used to identify the weight, so as to minimize the maximum absolute difference between the weight ratio and its corresponding comparison ([31, 32]). The model may produce multiple optimal solutions. Because the method is still relatively new, it is not commonly used in practical application. COPRAS was first proposed by [33]. It can use for multi-criteria evaluation of maximization and minimization of criteria values. This method considers the influence of maximization and minimization criteria on evaluation results respectively ([34]). This approach assumes that the importance and usefulness of the version under investigation are proportional to the standard system that adequately describes the alternatives and the value and weight of the criteria ([35]). The WASPAS approach is a combination of the Weighted Sum Model (WSM) and the Weighted Product Model (WPM) ([36]). This method was proposed by [37]. Because of its mathematical simplicity and ability to provide more precise results, it is now widely accepted as an effective decision-making tool. [38] proposed a new method to cope with multi-criteria decision problems, namely SECA. The purpose of this method is to identify both the overall performance score of the alternative and the weight of the criteria. [39] introduced a new method, MEREC, to determine the objective weight of criteria. This method adopts a novel criterion weighting idea. The method utilizes the removal effect of each criterion on the overall performance of alternatives to calculate the criterion weight. [40] proposed a multi-criteria inventory classification evaluation method based on Distance from Average Solution (EDAS). TOPSIS is a value-based compensation method proposed by [41], which orders alternatives according to their distance from two reference points, namely ideal (positive ideal) and the lowest point (negative ideal) alternatives. However, the TOPSIS method does not examine the relative importance of these distances, which is a limitation ([42]). Most of the above MCDM methods are relatively new, which provides an important reference for enriching MCDM methods. However, considering the universality of use, these methods are not used in this study. FCEM is based on fuzzy mathematics, applying the principle of fuzzy relation synthesis, quantifying some factors with unclear boundaries that are difficult to be quantified, and comprehensively evaluating the subjection level of the evaluated things from multiple factors. The advantages of FCEM method are: the mathematical model is simple and easy to master, especially the evaluation effect is better for multi-factor and multi-level complex problems, which are difficult to be replaced by other mathematical branches and models. Its characteristic lies in that the evaluation is carried out one by one, and has a unique evaluation value for the evaluated object, which is not subject to the object set of the evaluated object. This model is widely used, and the practical model using fuzzy comprehensive evaluation has achieved good economic and social benefits in many aspects.

The AHP, proposed by [43], is a method to assess the relative importance of projects based on the weight distribution of criteria. AHP is one of the earliest, most widely used and most mature MCDM methods. This method is easy to understand and easy to operate, which is also one of the reasons why this method is selected in this paper. AHP and FCEM are widely used in multi-criteria fuzzy synthesis, such as [44–46] and so on, all used AHP to calculate the weight of fuzzy comprehensive evaluation. [47] used FCEM and AHP methods to establish a comprehensive framework for evaluating the suitability of sustainable development of Lianyungang coastal reclamation project. The evaluation results are in good agreement with marine pollution data and water quality. [48] applied AHP and FCEM to train effectiveness evaluation engineering, constructed a standard system based on AHP theory, and introduced the concept of fuzzy optimization. The results show that this method is an effective method. To identify and classify risks and uncertainties in the soft beverage supply chain, [49] developed a fuzzy analytic hierarchy process (FAHP) and FCEM to evaluate these risks. The results show that the overall risk level of the soft drink supply chain is in the middle and low level, with the highest risk level of demand and the lowest risk level of infrastructure. [50] applied FCEM and AHP to evaluate the smart tourist attraction of HongShan Zoo, a popular tourist attraction in China. Based on the above advantages and characteristics of FCEM and AHP method and the universality of combining both methods, we also use FCEM and AHP method to study the priority of the layout of strategic emerging industries in Sichuan province.

## Materials and methods

### Decision model

As the decision-making mechanism of the layout of strategic emerging industries is a top-down process dominated by the government and supplemented by the market, it is necessary to follow such decision-making mechanism when establishing the selection model of strategic emerging industries. Assume that the set of industry choices made by a level is: *A* = (*a*_1_, *a*_2_, …, *a*_*n*_), *n* is the number of selected industries. Decision makers at this level need to make decisions on the spatial layout of *n* types of industries, and analyze the suitability of location conditions of subordinate regions at this level for alternative industries by assuming fixed industrial conditions, so that each type of alternative industries has a location priority selection order.

For example, for industry *a*_1_, the ranking of suitability of subordinate regions at this level to select this industry is $C_{a_{1}}=\left(c_{11},c_{12},\ldots ,C_{1m}\right)$, *m* is the number of subordinate regions. Therefore, the result of the industrial layout scheme of this level is represented by the matrix

$$X=\left[\begin{array}{cccc} c_{11} & c_{12} & \cdots & c_{1m}\\ c_{21} & c_{22} & \cdots & c_{2m}\\ \vdots & \vdots & \cdots & \vdots \\ c_{n1} & c_{n2} & \cdots & c_{nm} \end{array}\right].$$

The resources of each region are always limited, and it is impossible to distribute a certain alternative industry to all the subordinate regions of this level. Therefore, it is assumed that the region that each category of industry can be distributed is *i*, 1≤ *i* ≤ *m*. The number of industries that each region can choose to lay out is *j*, 1≤ *j* ≤ *n*.

For any industry *a*_*s*_, the decision scheme that the decision hierarchy arranges to deploy to the subordinate region is *Y* = [*c*_*s*1_, *c*_*s*2_, …, *c*_*s*i_], Industry *a*_*s*_ should be distributed to the subordinate areas of the hierarchy in an order of *c*_*1*_ to *c*_*i*_, with similar results for other industries. In practical decision making, is likely to appear this kind of circumstance, the subordinate area *c*_*i*_, condition is good, lead to all types of industries are chose to the region, more than the number of industries can decorate the area *j*, at this time will need to select the target area for all types of industries to sort, select one of the j industry, namely $Y_{c_{i}}=\left[a_{1i},\;a_{2i},\ldots a_{ji}\right]$. At the same time, due to the poor geographical conditions of some subordinate regions, no industry may choose to be arranged in this region, at this point, industries that are not fully arranged in *i* locations should be sorted and selected according to their suitability. It is assumed that the number of times subordinate region *c*_*r*_ is selected by industries is zero, and the set of all industries that have not arranged *i* locations is $A^{\prime }=\left[a_{1}^{'},\;a_{{}_{2}}^{'},\ldots ,a_{{}_{t}}^{'}\right]$ *t* is the number of industries. Therefore, the set of industry priority order that region *c*_*r*_ can choose is $Y_{c_{r}}=\left[a_{1r}{}^{\prime },\;a_{2r}{}^{\prime },\ldots ,a_{tr}{}^{\prime }\right]$, where the former *j* item is the industry that subordinate region *c*_*r*_ can choose to deploy.

### Evaluation method

#### Variables

The decision of the regional industry selection is made by the level of district and county. District and county decision-makers regard the location conditions as fixed values, analyze the layout conditions of different industries, and evaluate each alternative industry by selecting certain indicators, to further clarify the industry types that can be discussed at the level of district and county. When selecting evaluation indexes, regional factors are not considered, only the factors of the industry itself are considered. This paper selects the life cycle of the industry, the degree of mutual influence of the industry, the degree of industrial agglomeration, the effect of industrial development and the ability of industrial innovation as the evaluation index of the analysis and research. The establishment of the index system in this paper mainly refers to [51] research on the layout of strategic emerging industries in Beijing.

(1) The life cycle of the industry. Different districts and counties have different resource endowment conditions and industrial carrying capacity, so they put forward specific requirements for industries in different industrial life cycles. This paper reflects the life cycle of the industry by three indexes: the growth rate of industrial output value, the growth rate of fixed assets scale and the growth rate of industrial employment scale.

In different industrial life cycles, the total output value is constantly changing, usually rising, and then falling. The growth rate of industrial output value selected in this paper is the total output value of the current year compared with the total output value of the previous year. If the ratio is greater than 1, the total industrial output value increases, otherwise, the total output value decreases.


$$\text{Growth}\;\text{rate}\;\text{of}\;\text{industrial}\;\text{output}\;\text{value}=\frac{\text{gross}\;\text{industrial}\;\text{output}\;\text{value}\;\text{of}\;\text{the}\;\text{industry}\;\text{in}\;\text{the}\;\text{current}\;\text{year}}{\text{gross}\;\text{industrial}\;\text{output}\;\text{value}\;\text{of}\;\text{the}\;\text{industry}\;\text{in}\;\text{the}\;\text{previous}\;\text{year}}$$ (1)

Whether an industry has growth can be reflected by the growth and change of fixed assets scale. In general, firms increase investment in the early stages of industrial life, when fixed assets grow relatively fast. After reaching the mature stage, industrial investment is relatively stable, and the growth of fixed assets becomes gentle. Therefore, this index can be chosen to analyze the life stage characteristics of the industry.


$$\text{Growth}\;\text{rate}\;\text{of}\;\text{fixed}\;\text{assets}\;\text{scale}=\frac{\text{value}\;\text{of}\;\text{fixed}\;\text{assets}\;\text{in}\;\text{the}\;\text{current}\;\text{year}}{\text{value}\;\text{of}\;\text{fixed}\;\text{assets}\;\text{in}\;\text{the}\;\text{previous}\;\text{year}}$$ (2)

The principle of this indicator is the same as that of the above indicators, both of which grasp that the demand of the industry will be different in different life cycles. When the industry is in the growth stage, its demand for labor force is very strong.


$$\text{Growth}\;\text{rate}\;\text{of}\;\text{industrial}\;\text{employment}\;\text{scale}=\frac{\text{number}\;\text{of}\;\text{industrial}\;\text{employment}\;\text{in}\;\text{current}\;\text{year}}{\text{number}\;\text{of}\;\text{industrial}\;\text{employment}\;\text{in}\;\text{previous}\;\text{year}}$$ (3)

(2) Degree of interplay between industries. We know that everything in the world is universally connected, and the industrial development in the economic society is no exception. The development of the industry itself will not only have a certain impact on other related industries, but also is bound to be affected by the development of other industries. Usually we use influence coefficient and sensitivity coefficient to reflect this kind of impact and the degree of being affected ([52]).

Coefficient of influence represents the change degree of output level of other industries caused by every change of one unit in *i* industry. *A*_*ij*_ is the coefficient on row *i* and column *j* of the Leontief matrix (*I*−*A*)^−1^. Influence coefficient refers to the spread degree of production demand to all sectors of the national economy when a certain product sector adds a unit of final product. The greater the coefficient of influence, the greater the pulling effect of this sector on other sectors.


$$\text{Coefficient}\;\text{of}\;\text{influence}=E_{i}=\frac{\sum_{i=1}^{n}A_{ij}}{\frac{1}{n}\sum_{i=1}^{n}\sum_{j=1}^{n}A_{ij}}\left(i=1,\;2,\ldots ,n;\;j=1,\;2,\ldots ,n\right)$$ (4)

Coefficient of sensitivity is used to represent the change degree of output level of *i* industry caused by every change of one unit in *j* industry.


$$\text{Coefficient}\;\text{of}\;\text{sensitivity}=F_{i}=\frac{\sum_{j=1}^{n}A_{ij}}{\frac{1}{n}\sum_{i=1}^{n}\sum_{j=1}^{n}A_{ij}}\left(i=1,\;2,\ldots ,n;\;j=1,\;2,\ldots ,n\right)$$ (5)

(3) Degree of industrial agglomeration. The agglomeration of industry will bring the agglomeration economic benefit, that is, the high benefit produced by the concentration of production factors such as labor and capital. Industrial agglomeration can continuously absorb funds and talents from the outside, continuously export new products, and create more employment opportunities, which is what district and county level decision-makers want to see. This paper uses location entropy and spatial Gini coefficient to reflect the agglomeration degree of this industry.

Location entropy, also known as specialization rate, is used to measure the spatial distribution of elements in a region, reflect the degree of specialization of an industrial sector, and the status and role of a region in a high-level region. The calculation formula is

$$EI=\frac{q_{i}}{\sum_{i=1}^{n}q_{i}}/\frac{Q_{i}}{\sum_{i=1}^{n}Q_{i}}$$ (6)

Where, $q_{i}$ represents the output value level or number of employees of industry *i* in a region, and $\sum_{i=1}^{n}q_{i}$ represents the output value level or number of employees of all industries in the region. $Q_{i}$ represents the output value level or number of employees of all industries in the country, and $\sum_{i=1}^{n}Q_{i}$ represents the output value level or number of employees of all industries in the country.

Lorenz created a Lorenz curve to account for the average degree of social distribution when studying residents’ income distribution. Gini, based on Lorentz curve, proposed a statistical index to calculate the fairness of income distribution-Gini coefficient. Krugman et al. Constructed a spatial Gini coefficient to measure the degree of spatial distribution equilibrium of the industry by using the principles and methods of Lorentz curve and Gini coefficient. [53] et al. defined the spatial Gini coefficient when studying the measurement of manufacturing agglomeration degree in the United States, and the calculation formula is

$$G=\sum_{i}{\left(S_{i}-x_{i}\right)}^{2}$$ (7)

Where, G is the spatial Gini coefficient, *x*_*i*_ is the proportion of the number of employed persons in a region in the total number of employed persons in China, and *S*_*i*_ is the proportion of the number of employed persons in industry *i* in a region in the total number of employed persons in this industry in China. When *G* = 0, the spatial distribution of industries is uniform, the larger *G* (maximum value = 1) is, the higher the degree of industrial agglomeration in the region is.

(4) Industrial development effect. Whether an industry is suitable for development of a region depends on whether the industry can bring the necessary effects to regional development. This paper mainly studies the industrial development effect from three aspects: economic effect, environmental effect, and the social effect, and only one index is selected respectively to discuss.

Industry profit rate can reflect the profitability of the industry and whether it can create value for investors. Here, it is mainly uses to measure the economic benefits of an industry. In addition to this indicator, the total labor productivity can also be selected. The total labor productivity is the product production per unit time of each employee calculated according to the product value. It is the comprehensive expression of the enterprise’s production technology level, operation and management level, staff’s technical proficiency and labor enthusiasm ([54]).


Industryprofitrate=totalannualprofitoftheindustry/netassetinvestmentoftheindustry (8)

Energy consumption per unit added value reflects the amount of energy consumed by an industrial industry for every 1000 yuan of industrial added value.


$$\text{Energy}\;\text{consumption}\;\text{per}\;\text{unit}\;\text{added}\;\text{value}=\frac{\text{energy}\;\text{consumption}\;\text{of}\;\text{the}\;\text{industry}\;\left(\text{generally}\;\text{refers}\;\text{to}\;\text{tons}\;\text{of}\;\text{standard}\;\text{coal}\right)}{\text{industrial}\;\text{added}\;\text{value}\;\text{of}\;\text{the}\;\text{industry}}$$ (9)

Number of employees in unit added value links the industrial added value of the industry with the number of jobs, and measures how many jobs can be provided by the unit industrial added value, which reflects a social effect.


$$\text{Number}\;\text{of}\;\text{employees}\;\text{in}\;\text{unit}\;\text{added}\;\text{value}=\frac{\text{average}\;\text{number}\;\text{of}\;\text{employees}\;\text{in}\;\text{the}\;\text{industry}}{\text{annuel}\;\text{added}\;\text{value}\;\text{of}\;\text{the}\;\text{industry}}$$ (10)

(5) Industrial innovation capability. Innovation is the engine for the sustainable development of the industry. Only through continuous innovation can the industry accumulates wealth and develops gradually. In this paper, the proportion of R&D expenditure, the proportion of R&D personnel and the industrial added value per capita of R&D personnel are chosen to describe the innovation capability of the industry.


$$\text{Proportion}\;\text{of}\;\text{R}\&\text{D}\;\text{expenditure}=\frac{\text{annual}\;\text{R}\&\text{D}\;\text{expenditure}\;\text{of}\;\text{the}\;\text{industry}}{\text{total}\;\text{annual}\;\text{operating}\;\text{revenue}\;\text{of}\;\text{the}\;\text{industry}}$$ (11)

$$\text{Proportion}\;\text{of}\;\text{R}\&\text{D}\;\text{personnel}=\frac{\text{number}\;\text{of}\;\text{R}\&\text{D}\;\text{personnel}\;\text{in}\;\text{the}\;\text{industry}}{\text{average}\;\text{total}\;\text{number}\;\text{of}\;\text{employees}\;\text{in}\;\text{the}\;\text{industry}}$$ (12)

$$\text{Per}\;\text{capita}\;\text{industrial}\;\text{added}\;\text{value}\;\text{of}\;\text{R}\&\text{D}\;\text{personnel}=\frac{\text{added}\;\text{value}\;\text{of}\;\text{the}\;\text{industry}\;\text{in}\;\text{the}\;\text{year}}{\text{number}\;\text{of}\;\text{R}\&\text{D}\;\text{personnel}}$$ (13)

#### Methods

Fuzzy comprehensive evaluation method (FCEM) is a comprehensive evaluation method based on fuzzy mathematics. According to the membership theory of fuzzy mathematics, the comprehensive evaluation method transforms qualitative evaluation into quantitative evaluation, that is, fuzzy mathematics are used to make an overall evaluation of things or objects restricted by many factors. It has the characteristics of clear results and strong systematicness. It can better solve the fuzzy and difficult to quantify problems, and is appropriate for the solution of various uncertain problems.

Fuzzy comprehensive evaluation usually includes three aspects. Suppose there are *n* factors related to the evaluated thing, and we record it as *U* = {*u*_1_, *u*_2_, …,*u*_*n*_}, which is called the factor set. Suppose that all *m* possible comment sets, denoted by *V* = {*v*_1_, *v*_2_, …,*v*_*m*_}, are called judgment sets. Due to the different status and functions of each factor, it is usually considered to be measured by weight, which is recorded as *A* = {*a*_1_, *a*_2_, …,*a*_*n*_}.

For the factor set weight vector *A* = {*a*_1_, *a*_2_, …,*a*_*n*_}, this paper uses AHP to determine it. AHP consists of the following five steps

1. Determine the research object and target;
2. Set the hierarchy according to the factors of the target;
3. Establish judgment matrix and solve it;
4. Consistency inspection; According to the solution of the eigenvalue problem of matrix A, $\text{A}W=\lambda_{\max}W$, normalization is carried out: $CI=\frac{\lambda_{\max}-n}{n-1}$, Then calculate the random consistency ratio $CR=\frac{CI}{RI}$, *RI* is the average random consistency index. When CR<0.10, it can be considered that the test has passed and the weight calculation result is valid.
5. According to the calculation results, determine the weight of the index system.

The methods and steps of fuzzy comprehensive evaluation are briefly introduced below

1. Deterministic factor set *U* = {*u*_1_, *u*_2_, …,*u*_*n*_};
2. Determine the evaluation set *V* = {*v*_1_, *v*_2_, …,*v*_*m*_};
3. *r*_*i*_ = {*r*_*i*1_, *r*_*i*2_, …,*r*_*im*_} obtained by single factor evaluation;
4. Construct comprehensive evaluation matrix
$$R=\left[\begin{array}{cccc} r_{11} & r_{12} & \cdots & r_{1m}\\ r_{21} & r_{22} & \cdots & r_{2m}\\ \cdots & \cdots & \cdots & \cdots \\ r_{n1} & r_{n2} & \cdots & r_{nm} \end{array}\right]$$
5. Comprehensive evaluation: For weight *A* = {*a*_1_, *a*_2_, …,*a*_n_}, calculate *B* = *A* ◦ *R* and make judgment according to the princple of maximum membership degree.

#### Research steps

1. Determining evaluation factors, Set *U* = {*u*_1_, *u*_2_, …,*u*_*m*_} to represent *m* evaluation indexes of the evaluated object. Since five evaluation indexes are determined in this paper, *U* = {*u*_1_, *u*_2_, …,*u*_*5*_}, *u*_1_ is the life cycle of the industry, *u*_2_ is the degree of industrial interaction, *u*_3_ is the degree of industrial agglomeration, *u*_4_ is the effect of industrial development, and *u*_5_ is the ability of industrial innovation.
2. Determination of evaluation grade, *S* = {*s*_1_, *s*_2_, …,*s*_*n*_} is the possible evaluation level of each index factor. Four grades are determined in this paper, *S* = {*s*_1_, *s*_2_, *s*_3_, *s*_*4*_} means *S* = {Not suitable, Suitable, More suitable, Very suitable}.
3. Construct the evaluation matrix and determine the weight. Firstly, the single-factor evaluation is carried out. Starting from *u*_*m*_ (*m* = 1, 2, …, 5), the membership degree of each evaluation grade *s*_*j*_ (*j* = 1, 2, 3, 4) is *r*_*mj*_, so the single-factor evaluation set *r*_*m*_ = (*r*_*m*1_, *r*_*m*2_, *r*_*m*3_, *r*_*m*4_) can be obtained. The fuzzy relation R from U to S of each evaluated object can be obtained by combining five first-level evaluation indexes, i.e.

$$R={\left(r_{mj}\right)}_{5\times 4}=\left[\begin{array}{cccc} r_{11} & r_{12} & r_{13} & r_{14}\\ r_{21} & r_{22} & r_{23} & r_{24}\\ \cdots & \cdots & \cdots & \cdots \\ r_{51} & r_{52} & r_{53} & r_{54} \end{array}\right].$$

**Fig 1:**
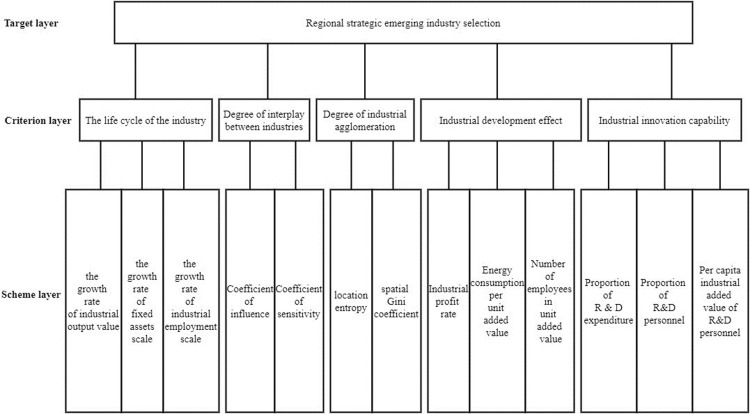
Analytic hierarchy process structure diagram.

Combined with the above established secondary evaluation index system, the following hierarchy analysis structure table (Table 1) can be obtained. The hierarchical analysis structure can also be represented by a figure, as shown in Fig 1.

**Table 1:** Hierarchy analysis structure table.

Target layer	Criterion layer	Scheme layer
Regional strategic emerging industry selection model (*A*)	The life cycle of the industry (*u*_1_)	the growth rate of industrial output value (*u*_11_)
		the growth rate of fixed assets scale (*u*_12_)
		the growth rate of industrial employment scale (*u*_13_)
	Degree of interplay between industries (*u*_2_)	Coefficient of influence (*u*_21_)
		Coefficient of sensitivity (*u*_22_)
	Degree of industrial agglomeration (*u*_3_)	location entropy (*u*_31_)
		spatial Gini coefficient (*u*_32_)
	Industrial development effect (*u*_4_)	Industrial profit rate (*u*_41_)
		Energy consumption per unit added value (*u*_42_)
		Number of employees in unit added value (*u*_43_)
	Industrial innovation capability (*u*_5_)	Proportion of R & D expenditure (*u*_51_)
		Proportion of R&D personnel (*u*_52_)
		Per capita industrial added value of R&D personnel (*u*_53_)

The weight of the criterion layer to the target layer is *W*_*A*_. According to the *AHP*, *W*_*A*_ is required to construct the paired comparison matrix *A* of the criterion layer first. When comparing the importance of the *i*-th element and the *j*-th element relative to a factor in the upper layer, it is described by the quantitative relative weight *a*_*ij*_. if there are *n* elements involved in the comparison, *A* = (*a*_*ij*_)_n × n_ is called the paired comparison matrix. The value of *a*_*ij*_ in the pairwise comparison matrix can be assigned according to the scale in Table 2 below with reference to [55]’s proposal. *a*_*ij*_ is taken in the middle of 1–9 and its reciprocal.

**Table 2:** Scale and meaning of pairwise comparison matrix.

Symbol	Meanings
*a*_*ij*_ = 1	Element *i* and element *j* are of the same importance to the factors of the previous level
*a*_*ij*_ = 3	Element *i* is slightly more important than element *j*
*a*_*ij*_ = 5	Element *i* is more important than element *j*
*a*_*ij*_ = 7	Element *i* is much more important than element *j*
*a*_*ij*_ = 9	Element *i* is quite important compared to element *j*
*a*_*ij*_ = 2*n*, *n =* 1, 2 3, 4	The importance of element *i* over element *j* is between *a*_*ij*_ = 2*n* − 1 and *a*_*ij*_ = 2*n* + 1

Characteristics of pairwise comparison matrices, $a_{ij}>0,\;a_{ij}=\frac{1}{a_{ji}}$, when *i* = *j*, *a*_*ij*_ = 1.

According to this principle, this paper constructs the following pairwise comparison matrix *A* of criterion layer factors relative to the target layer

$$A=\left[\begin{array}{ccccc} 1 & \frac{1}{2} & \frac{1}{3} & \frac{1}{4} & \frac{1}{4}\\ 2 & 1 & \frac{1}{2} & \frac{1}{3} & \frac{1}{4}\\ 3 & 2 & 1 & \frac{1}{2} & \frac{1}{3}\\ 4 & 3 & 2 & 1 & \frac{1}{2}\\ 4 & 4 & 3 & 2 & 1 \end{array}\right].$$

The maximum eigenvalue *λ*_max_ = 5.1022 of the matrix. $CI=\frac{\lambda_{max}-n}{n-1}=\frac{5.1022-5}{5-1}=0.0225$, which measures the degree of inconsistency of the matrix *A*.

According to relevant data, the average random consistency index *RI* = 1.0959 and random consistency ratio $CR=\frac{CI}{RI}=\frac{0.0255}{1.0959}=0.0233<0.1$ of *A* can be obtained, so the inconsistency degree of *A* is acceptable. The eigenvector corresponding to the maximum eigenvalue is [0.1257, 0.1869, 0.3075, 0.5037, 0.7753].

Therefore, *W*_*A*_ = [0.0662, 0.0984, 0.1619, 0.2652, 0.4082].

Similarly, $\mu_{1}=\left[\begin{array}{ccc} 1 & 3 & 2\\ 1/3 & 1 & 1/2\\ 1/2 & 2 & 1 \end{array}\right]$, *λ*_max_ = 3.0092, *CI* = (*λ*_max_− 3) / 2 = 0.0046, After investigation, *RI* = 0.58, *CR* = *CI* / *RI* = 0.0079 < 0.1, so $W_{u_{1}}=[0.5396,0.1634,0.2969]$.

$u_{2}=\left[\begin{array}{cc} 1 & 4\\ 1/4 & 1 \end{array}\right]$, *λ*_max_ = 2, *CI* = 0, *RI* = 0 < 0.1, $W_{u_{2}}=[0.8000,0.2000]$. $u_{3}=\left[\begin{array}{cc} 1 & 3\\ 1/3 & 1 \end{array}\right]$, *λ*_max_ = 2, *CR* = 0 < 0.1, $W_{u_{3}}=[0.7500,0.2500]$.


$$u_{4}=\left[\begin{array}{ccc} 1 & 1/5 & 1/4\\ 5 & 1 & 3\\ 4 & 1/3 & 1 \end{array}\right],\lambda_{max}=\;3.0858,CR=\;0.0740\;<\;0.1,W_{u_{4}}=[0.0936,0.6267,0.2797].$$

$$u_{5}=\left[\begin{array}{ccc} 1 & 4 & 2\\ 1/4 & 1 & 1/3\\ 1/2 & 3 & 1 \end{array}\right],\lambda_{max}=\;3.0183,CR=\;0.0158\;<\;0.1,W_{u_{5}}=\left[0.5584,0.1219,0.3196\right].$$

Therefore, it can be concluded that the weight of each scheme layer relative to the target layer is *W*_*A*_ = [0.0357, 0.0161, 0.0197, 0.0787, 0.0197, 0.1214, 0.0405, 0.0248, 0.1662, 0.0742, 0.2279, 0.0498, 0.1305]

Delphi Method is a structured decision support technology. Its purpose is to obtain relatively objective information, opinions, and perspectives through independent and repeated subjective judgments of several experts in the process of information collection. The characteristic of the Delphi method is that the experts whose opinions are sought to express their opinions anonymously, and the experts cannot discuss with each other and do not have horizontal contact, so as to avoid the convergence of expert opinions to a few influential experts. In this paper, the standard value of each index is determined by the Delphi method, that is, after scoring by experts, membership function is established after sorting. In this paper, 30 experts from relevant government departments, industry and academia were invited to score the standard values of the selected evaluation indicators of strategic emerging industries through emails and letters. Three rounds of surveys were conducted, and 18 valid survey data were finally obtained.

Not suitable (*s*_1_)

$$r_{i1}=\left\{\begin{array}{c} 1,x\leq a_{1}\\ \left(a_{2}-x\right)/\left(a_{2}-a_{1}\right),a_{1}<x<a_{2}\\ 0,x\geq a_{2} \end{array}\right.$$

Suitable (*s*_2_)

$$r_{i2}=\left\{\begin{array}{c} \left(a_{3}-x\right)/\left(a_{3}-a_{2}\right),a_{2}<x<a_{3}\\ \left(x-a_{1}\right)/\left(a_{2}-a_{1}\right),a_{1}<x<a_{2}\\ 0,x\leq a_{1},x\geq a_{3} \end{array}\right.$$

More suitable (*s*_3_)

$$r_{i3}=\left\{\begin{array}{c} \left(a_{4}-x\right)/\left(a_{4}-a_{3}\right),a_{3}<x<a_{4}\\ \left(x-a_{2}\right)/\left(a_{3}-a_{2}\right),a_{2}<x<a_{3}\\ 0,x\leq a_{2},x\geq a_{4} \end{array}\right.$$

Very suitable (*s*_4_)

$$r_{i4}=\left\{\begin{array}{c} 0,x\leq a_{3}\\ \left(x-a_{3}\right)/\left(a_{4}-a_{3}\right),a_{3}<x<a_{4}\\ 1,x\geq a_{4} \end{array}\right.$$

Where, the value of *a*_1_, *a*_2_, *a*_3_, *a*_4_ is as follows (Table 3).

**Table 3:** Critical value of membership degree of each evaluation index.

standard	*a*_1_	*a*_2_	*a*_3_	*a*_4_
growth rate of industrial output value	0.95	1.08	1.2	1.3
Growth rate of fixed assets scale	0	1	1.1	1.25
Growth rate of industrial employment scale	0	1	1.1	1.2
Coefficient of influence	0.6	1	1.1	1.3
Coefficient of sensitivity	0.6	1	2	2.5
Location entropy	1.2	1.6	2	2.5
Spatial Gini coefficient	0.0004	0.001	0.002	0.004
Industrial profit rate	0.05	0.1	0.2	0.3
Energy consumption per unit added value	0.8	1	1.5	2
Number of employees in unit added value	1	3	5	10
Proportion of R&D expenditure	0.005	0.01	0.02	0.05
Proportion of R&D personnel	0.02	0.05	0.1	0.2
Per capita industrial added value of R & D personnel	150	200	350	500

## Results

### Data acquisition and processing

This paper selects the input-output table of 42 departments in Sichuan province in 2012 (the data are from the official website of the Sichuan Provincial Bureau of Statistics) as the basic data for the calculation of relevant indicators. Limited to space, only the coefficient of sensitivity and coefficient of influence are listed here. The calculation results are given in table in S1 Appendix.

What we want to analyze is the influence coefficient and sensitivity coefficient of strategic emerging industries. Therefore, we need to connect 42 departments with strategic emerging industries. A class of strategic emerging industries may involve multiple departments. Here, the average method is used to get the coefficient of influence and coefficient of sensitivity of corresponding strategic emerging industries. The calculation results are presented in Table 4.

**Table 4:** Coefficient of influence and coefficient of sensitivity of strategic emerging industries.

Industry	Coefficient of influence	Coefficient of sensitivity
NS&EPI	1.0849	0.7721
BI	1.1301	1.1118
NMI	1.1837	1.1759
NEI	1.1428	1.1854
HEEMI	1.2257	0.6529
NGITE	1.1653	0.7085
NEVI	1.2314	0.7175

Where, NGITE: New generation information technology industry; NEVI: New energy vehicle industry; HEEMI: High-end equipment manufacturing industry; NEI: New energy industry; BI: Biological industry; NS&EPI: Energy saving and environmental protection industry; NMI: New material industry. The same goes for the following.

**Fig 2:**
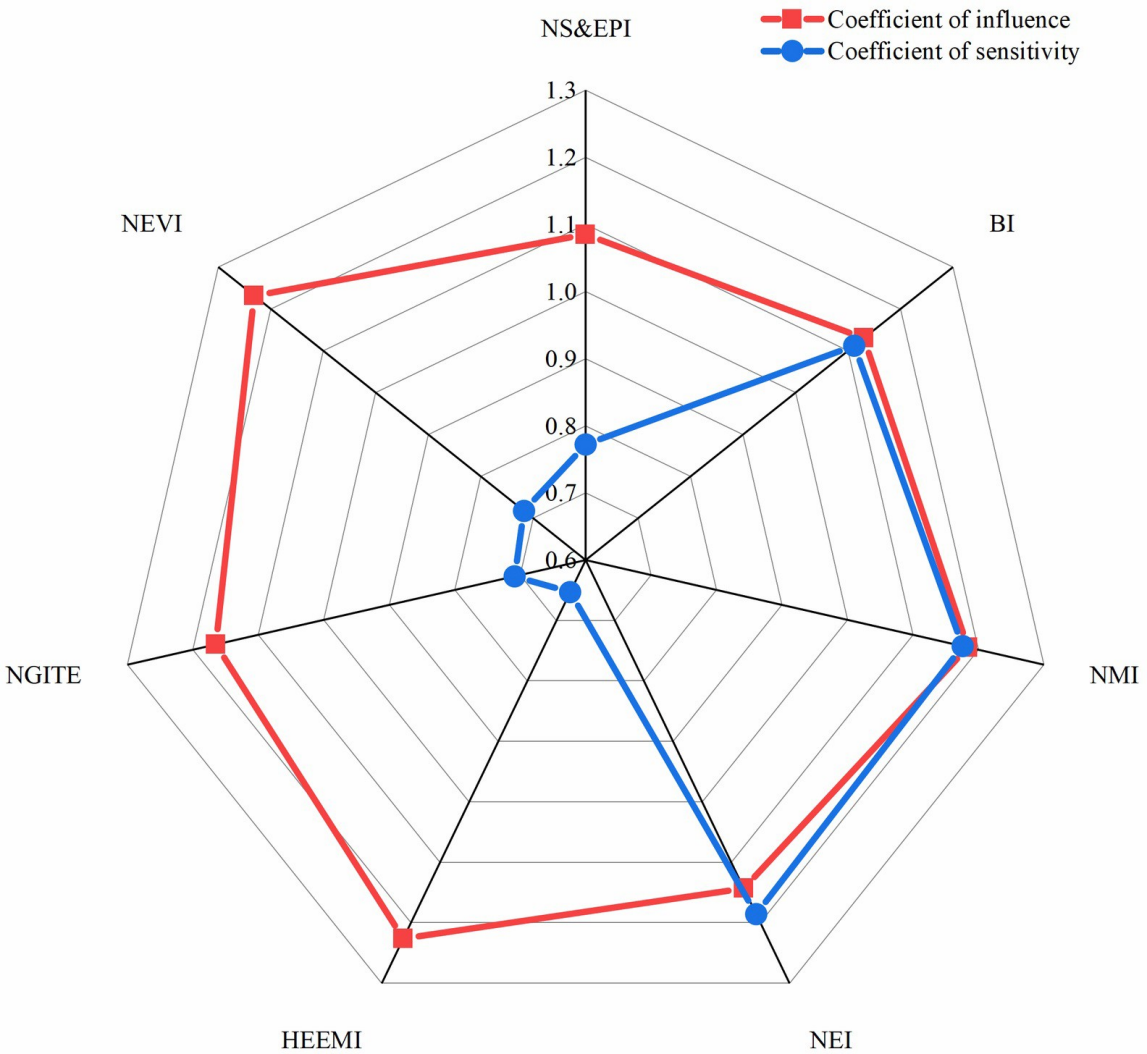
Coefficient of influence and coefficient of sensitivity of each industry.

According to Fig 2, it can be clearly seen that NEVI industry have the highest coefficient of influence, while NEI and NMI industry has high coefficient of sensitivity. This indicates that the NEVI industry has a great impact on other related industries, and the industry may be in the upstream of the industry. However, NEI and NMI industries are greatly affected by their related industries and are in the lower reaches of the industry.

For other relevant indicators, calculate the data of 42 departments according to the statistical yearbook, and then calculate the relevant indicators of strategic emerging industries through weighted average and other methods according to the relationship between strategic emerging industries and 42 departments.

### Development layout of strategic emerging industries

Using the data of China’s industrial enterprise database, China’s industrial economic statistical yearbook, Sichuan statistical yearbook, Sichuan Science and technology statistical yearbook and other data, the corresponding evaluation index values of the seven categories of strategic emerging industries selected in this paper are calculated. The calculation results are shown in Table 5.

**Table 5:** Evaluation results of strategic emerging industries selection.

Index system	NS&EPI	BI	NEI	HEEMI	NGITE	NMI	NEVI
*u*_11_	1.0585	0.9970	1.0944	0.9806	1.0756	0.9805	1.0583
*u*_12_	1.0128	1.0019	1.0042	1.1987	1.1878	1.0009	1.0099
*u*_13_	1.0709	1.0070	1.1496	0.9803	1.0788	1.0261	1.0360
*u*_21_	1.0849	1.1301	1.1837	1.1428	1.2257	1.1653	1.2314
*u*_*2*2_	0.7721	1.1118	1.1759	1.1854	0.6529	0.7085	0.7175
*u*_31_	1.7889	1.5465	1.5537	1.3133	1.6207	1.7972	1.8554
*u*_32_	0.0039	0.0013	0.0009	0.0006	0.0013	0.0016	0.0005
*u*_41_	0.1325	0.1156	0.0689	0.1211	0.0879	0.0854	0.1572
*u*_42_	1.0582	1.3428	1.3524	0.5391	0.7318	1.0823	0.6812
*u*_43_	3.6722	3.9326	2.7660	3.1288	4.8367	5.4877	2.9429
*u*_51_	0.0090	0.0082	0.0065	0.0116	0.0123	0.0072	0.0121
*u*_52_	0.0331	0.0389	0.0357	0.0340	0.0578	0.0336	0.0348
*u*_53_	280.8123	321.9320	506.0854	255.2974	264.0095	162.1386	328.5579

Taking the above calculation results into the corresponding membership function, the membership of each evaluation index of each industry to the evaluation set can be obtained, and seven evaluation matrices R can be obtained, as shown in Table in S2 Appendix.

Based on this, this paper uses the weight *W*_*A*_ calculated earlier to multiply the above seven R matrices with *W*_*A*_ to obtain the evaluation result matrix of seven industries. After normalization, it is shown in Table 6.

**Table 6:** Industrial evaluation result matrix table.

	Not suitable	Suitable	More suitable	Very suitable
NS&EPI	0.0903	0.4811	0.3903	0.0383
BI	0.1388	0.5712	0.2783	0.0117
NEI	0.2270	0.4881	0.1126	0.1723
HEEMI	0.1674	0.4752	0.1647	0.1926
NGITE	0.0242	0.4993	0.2526	0.2239
NMI	0.3010	0.3197	0.3465	0.0327
NEVI	0.0806	0.4170	0.2856	0.2168

In order to further make a reasonable decision, this paper chooses to assign a value to the corresponding judgment (Table 7).

**Table 7:** Judge statement assignment.

	Not suitable	Suitable	More suitable	Very suitable
Assignment	2	4	6	8

**Fig 3:**
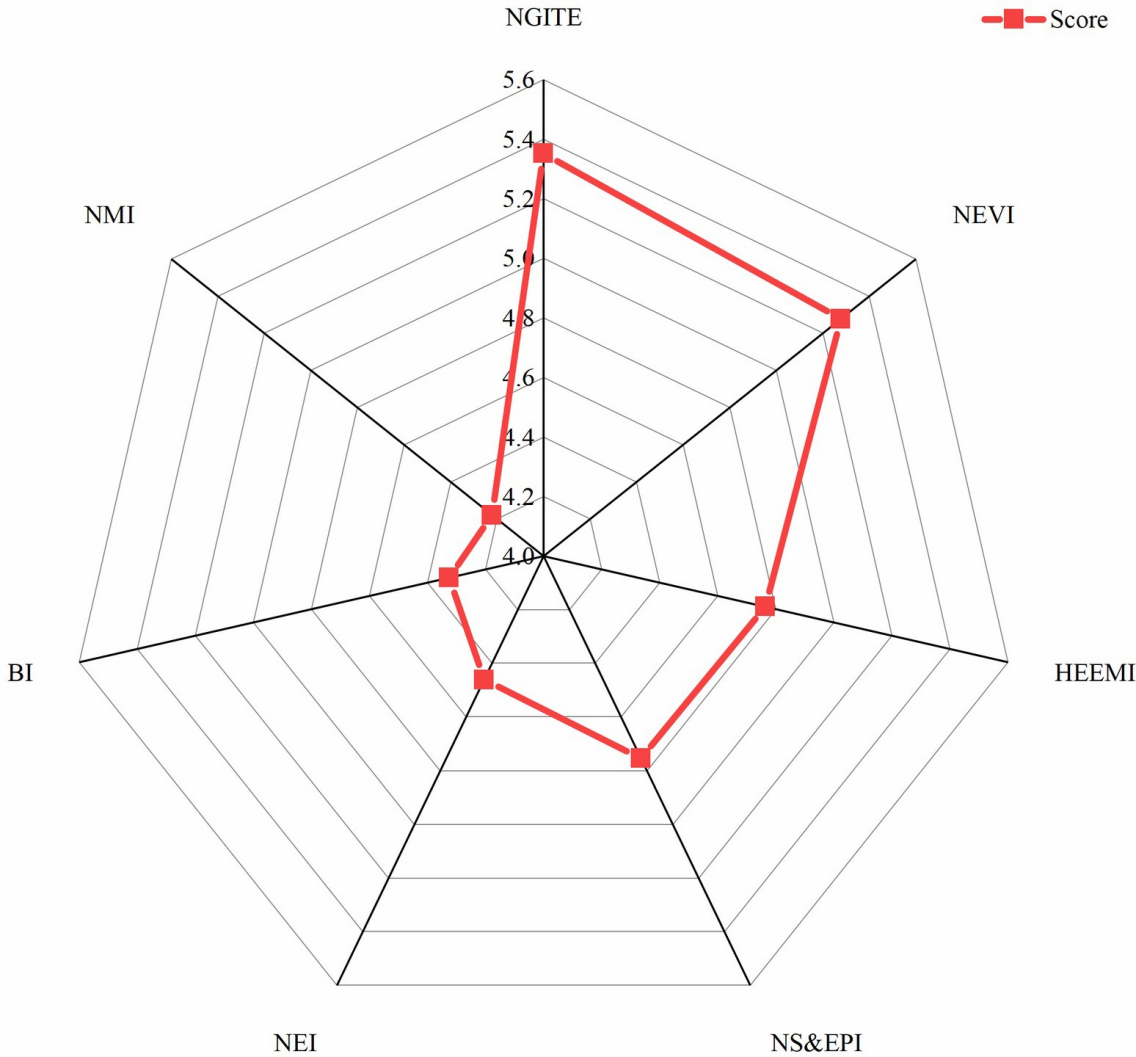
Comparison of comprehensive scores of various industries.

Therefore, the score of weighted average comprehensive evaluation is finally calculated as the basis for industrial selection. The results are shown in Table 8 and Fig 3.

**Table 8:** Industrial comprehensive score table.

Industry	Score
NGITE	5.3524
NEVI	5.2772
HEEMI	4.7646
NS&EPI	4.7532
NEI	4.4604
BI	4.3258
NMI	4.2214

**Fig 4:**
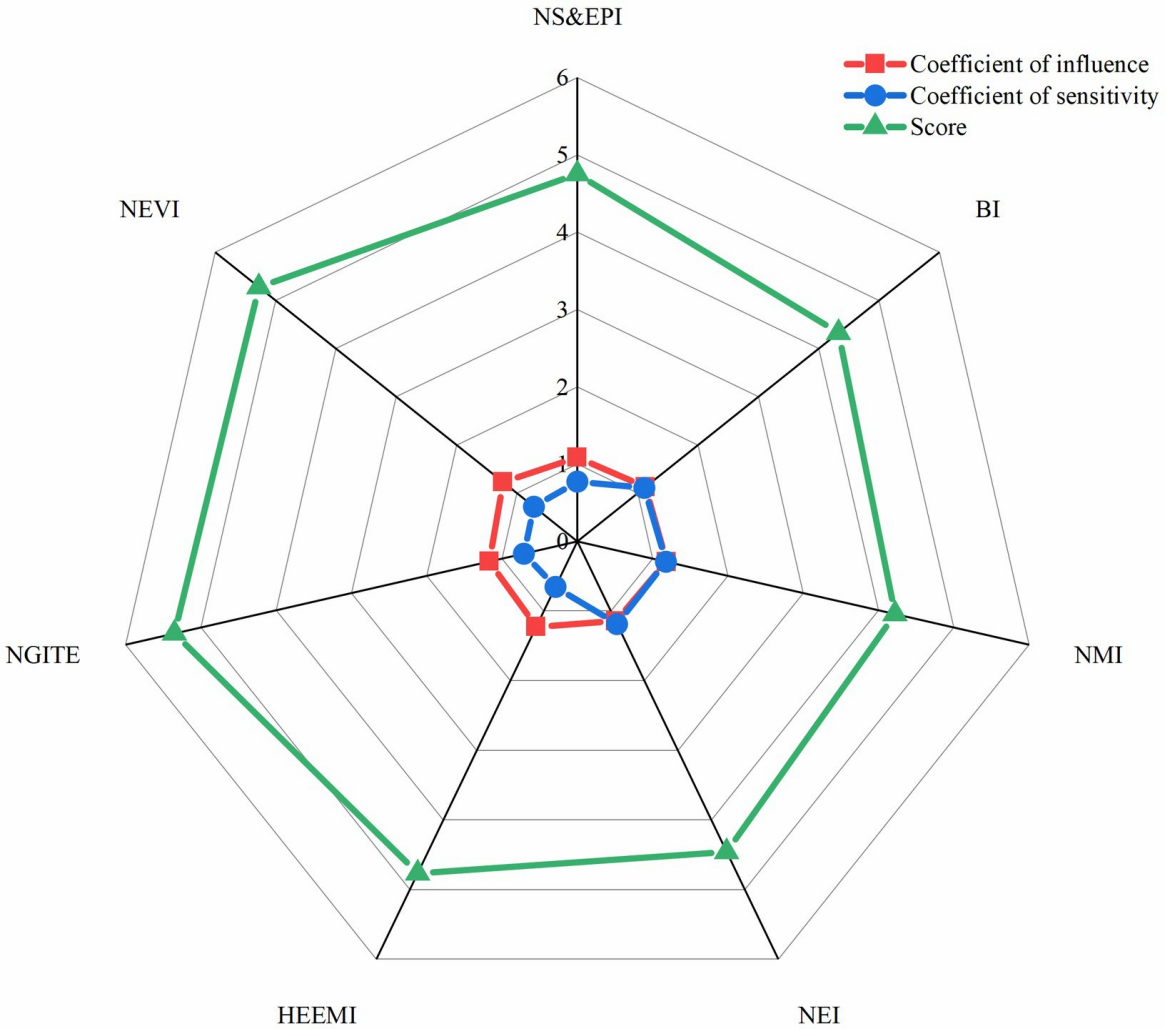
Coefficient of Influence, coefficient of sensitivity and comprehensive score.

The order of industrial types listed in Table 8 according to the comprehensive score is the priority of strategic emerging industries in Sichuan province obtained in this paper. This paper holds that Sichuan province should be a priority for the development of new generation information technology industry and new energy vehicle industry, followed by high-end equipment manufacturing, energy conservation and environmental protection, new energy, and other industries. Grasp the development opportunities of information technology upgrading and industrial integration, expand and strengthen advantageous industries by breaking through high-end links and key technologies, cultivate new business forms, improve basic products, realize the transformation of the province’s new generation of information technology industry from large to strong, form an industrial cluster with strong competitiveness, and seize the commanding height of the development of information technology industry. Fig 4 shows that the coefficient of influence and coefficient of sensitivity have different influences on the comprehensive score of strategic emerging industries. Specifically, the larger the coefficient of influence is, the higher the comprehensive score is, while the larger the coefficient of sensitivity is, the lower the comprehensive score is. The reason for this is inextricably linked to the concepts of coefficient of influence and coefficient of sensitivity. The coefficient of influence is the degree to which an industry influences other industries, while the coefficient of sensitivity is the extent to which an industry is influenced by other industries. This also confirms the rationality of our choice of these two indicators to choose the layout of strategic emerging industries.

## Discussion

### Spatial distribution status

This paper uses industry codes to classify strategic emerging industries and national economic industries to establish a corresponding relationship. Although it cannot be guaranteed to be completely correct, it is considered that this is adequate for empirical analysis. During the 12th Five Year Plan period, Sichuan province scientifically selected strategic emerging industries such as new generation information technology industry, new energy, high-end equipment manufacturing, new materials, biology, energy conservation and environmental protection, and achieved fruitful results in recent years, forming a certain strategic emerging industrial cluster with competitive advantages, becoming an important new generation information technology, high-end equipment manufacturing, new materials Energy conservation and environmental protection industry base. In 2015, the total output value of strategic emerging industries was 567.15 billion yuan, 2.5 times that of 2010; Accounting for 13.9% of the total output value of industries above Designated Size in the province, an increase of 4.5 percentage points over 2010. When studying the layout of strategic emerging industries in Sichuan province, this paper focuses on the seven categories of industries announced by the state.

This paper selects industrial enterprise data of Sichuan province from 2009 to 2013 for research. The total data from 2009 to 2013 is about 50000. These data mainly include enterprise address coordinates, industry code, main products, and main financial index information. According to the relationship between national economy industry code and strategic emerging industry code, use Excel VLOOKUP to find all data points related to strategic emerging industries, and finally screen out about 25000 sample points. Taking the prefecture level as the unit, the number of enterprises, industrial types, industrial added value, operating income, and employees at the end of the year of strategic emerging industries involved in each prefecture and city in Sichuan province are summarized to obtain the statistical data of each prefecture and city by industrial category, which is used as the basis for analysis. Taking the data of 2013 as an example, 5460 data points were selected in these sample enterprises, including 268 new generation information technology enterprises, 561 new energy automobile enterprises, 504 high-end equipment manufacturing enterprises, 330 new energy industry enterprises, 931 biological industry enterprises, 1296 energy conservation and environmental protection industry enterprises and 1570 new materials industry enterprises.

According to the detailed name and registered address of the enterprise at the sample point, XGeocoding_v2 is used. Geographic coordinate conversion function of XGeocoding_v2 software captures the address coordinates of the sample point enterprise and converts them into WGS84 longitude and latitude coordinate system, the sample points are imported into ArcGIS software to generate its space vector layer.

#### Analysis of the number of enterprises

**Fig 5:**
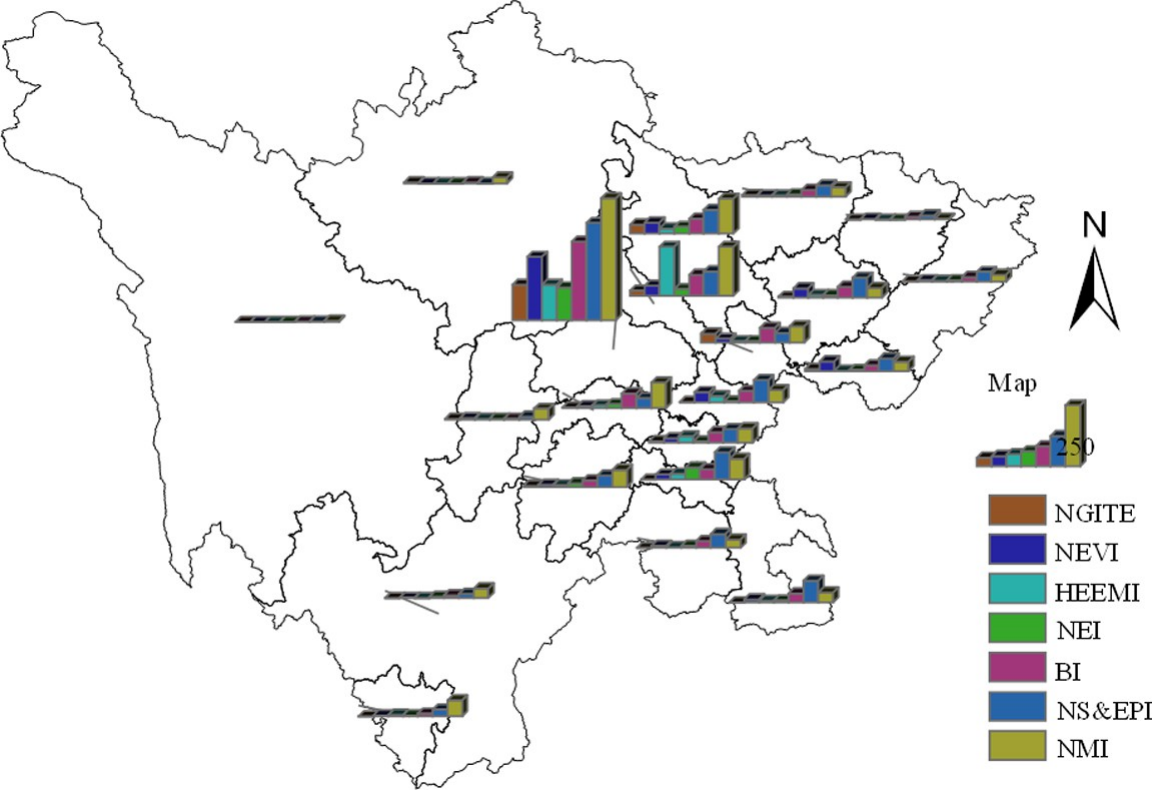
Number distribution of strategic emerging industries by industry in Sichuan province. The bottom map data from the website: http://datav.aliyun.com/portal/school/atlas/area_selector. The source data is from Autonavi Open platform.

The 5460 sample point enterprises were summarized by industry and city location, and ARCGIS software was used to generate a bar chart, as showed in Fig 5. It can be seen from the analysis results that the number of enterprises in the new material industry, biological industry and energy conservation and environmental protection industry accounts for a large proportion, while the number of enterprises in the new generation information technology industry, new energy vehicle industry, High-end equipment manufacturing industry and new energy industry accounts for a relatively small proportion. From the perspective of spatial distribution of Sichuan province, strategic emerging industries in Sichuan province are concentrated in Chengdu Plain Economic Zone (Ya’an City, Leshan City, Meishan City, Ziyang City, Mianyang City, Deyang City and Chengdu), southern Sichuan Economic Zone (Neijiang City, Zigong City, Luzhou City and Yibin City), and Northeast Sichuan Economic Zone (Guangyuan City, Bazhong City, Nanchong City, Dazhou city and Guang’an City) The number of strategic emerging industry enterprises is relatively small. Of course, the proportion of strategic emerging industry enterprises is less in Panxi Economic Zone (Liangshan Prefecture and Panzhihua City) and Northwest Sichuan Economic Zone (Ganzi Prefecture and Aba Prefecture), of which Northwest Sichuan Economic Zone is the region with the least distribution of strategic emerging industries in Sichuan province. Among the Chengdu Plain Economic Zone with a largest number of strategic emerging industrial enterprises, Chengdu has the largest number of strategic emerging industrial enterprises and the most comprehensive industrial type layout, which basically form the situation of "one dominant" in Chengdu.

From the above analysis, it can be seen that the number of enterprises in the new generation of information technology industry and new energy vehicle industry recommended for priority development is not dominant, the foundation of industrial development is still relatively weak, and there is a situation of "one dominant" in regional distribution. Therefore, current development priorities of strategic emerging industries in Sichuan province may not match the regional advantages.

#### Analysis of total industrial output value

**Fig 6:**
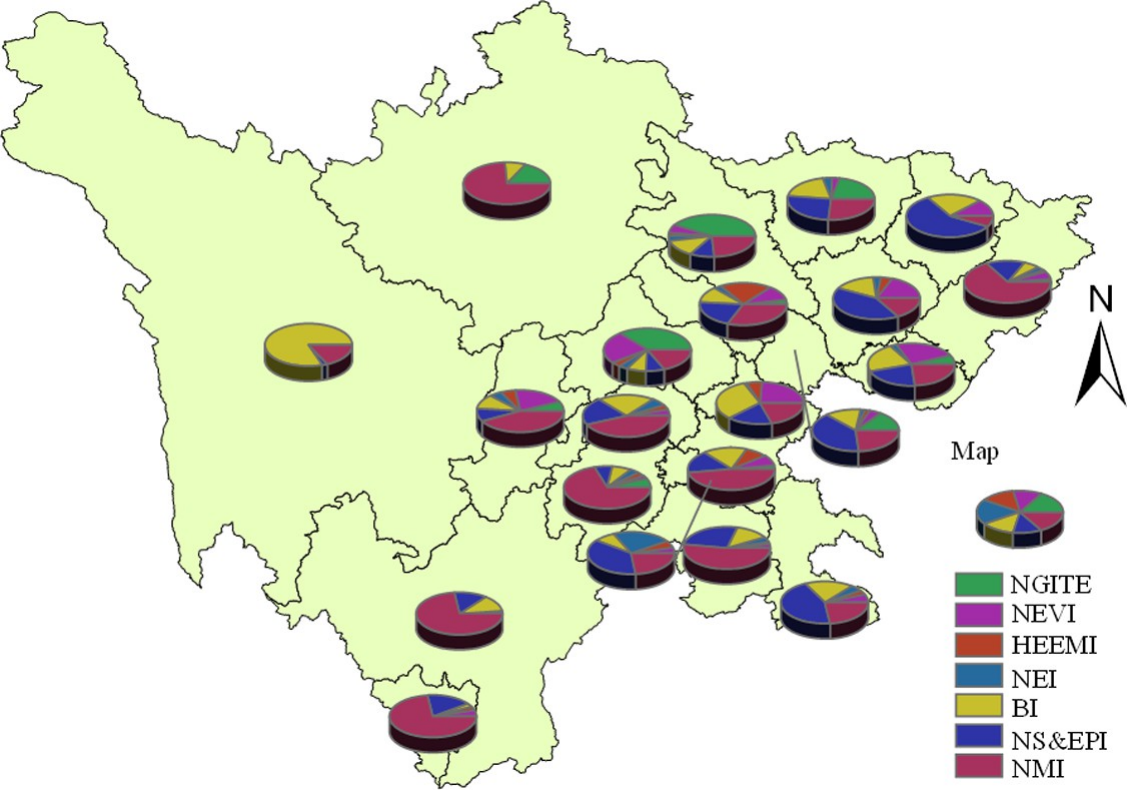
Distribution of total industrial output value by industry at the sample points of strategic emerging industries in Sichuan province. The bottom map data from the website: http://datav.aliyun.com/portal/school/atlas/area_selector. The source data is from Autonavi Open platform.

The total industrial output value of enterprises in the sample points is classified and summarized according to local and municipal industries, and its layout is illustrated in Fig 6. Among them, the new generation of information technology industry is a knowledge intensive industry, which has high requirements for talents and scientific and technological resources, and its total industrial output value accounts for a relatively high proportion in the Chengdu Plain Economic Zone. Biological industry and new material industry are arranged in the five economic zones, and biological industry contributes the most to the output value of Northwest Sichuan Economic Zone. The high proportion of the total output value of southern Sichuan Economic Zone is mainly the new material industry and energy conservation and environmental protection industry. The high-end equipment manufacturing industry is focused on old industrial bases such as Chengdu and Deyang. In terms of the total industrial output value of the industry, the new generation of information technology industry and the new energy vehicle industry have not formed an obvious scale advantage, which is related to the current strategic emerging industry development focus.

#### Analysis on the number of employees

**Fig 7:**
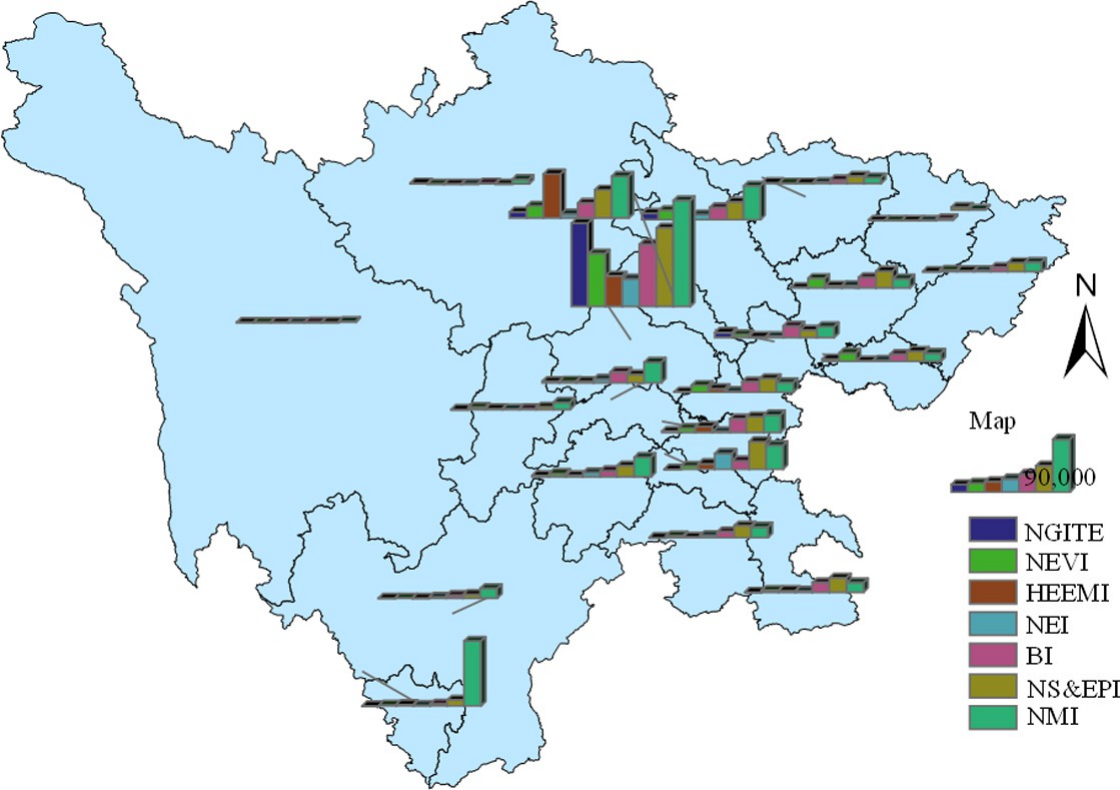
Number distribution of employees in sample points of strategic emerging industries. The bottom map data from the website: http://datav.aliyun.com/portal/school/atlas/area_selector. The source data is from Autonavi Open platform.

Analyze the employees in various regions and industries of the sample enterprises, and summarize the data of employees in different regions and industries, as showed in Fig 7. It can be seen that the distribution law of the number of employees is very similar to the number of enterprises and total industrial output value. Panxi Economic Zone, Northwest Sichuan ecological economic zone and Northeast Sichuan economic zone are less distributed, among which the Northwest Sichuan ecological economic zone has the least distribution of employees. There are a lot of employees in Chengdu Plain Economic Zone and southern Sichuan Economic Zone. In terms of the total number of employees, Chengdu Plain Economic Zone has a very high concentration and is outstanding in solving people’s livelihood problems such as regional employment. A good industrial employment situation will promote the continuous development of the industry. At present, the new generation of information technology industry and new energy automobile industry in Sichuan province have shown a positive trend in promoting employment. This shows the correctness and rationality of the development priority plan for strategic emerging industries we put forward.

#### Analysis of annual operating revenue

**Fig 8:**
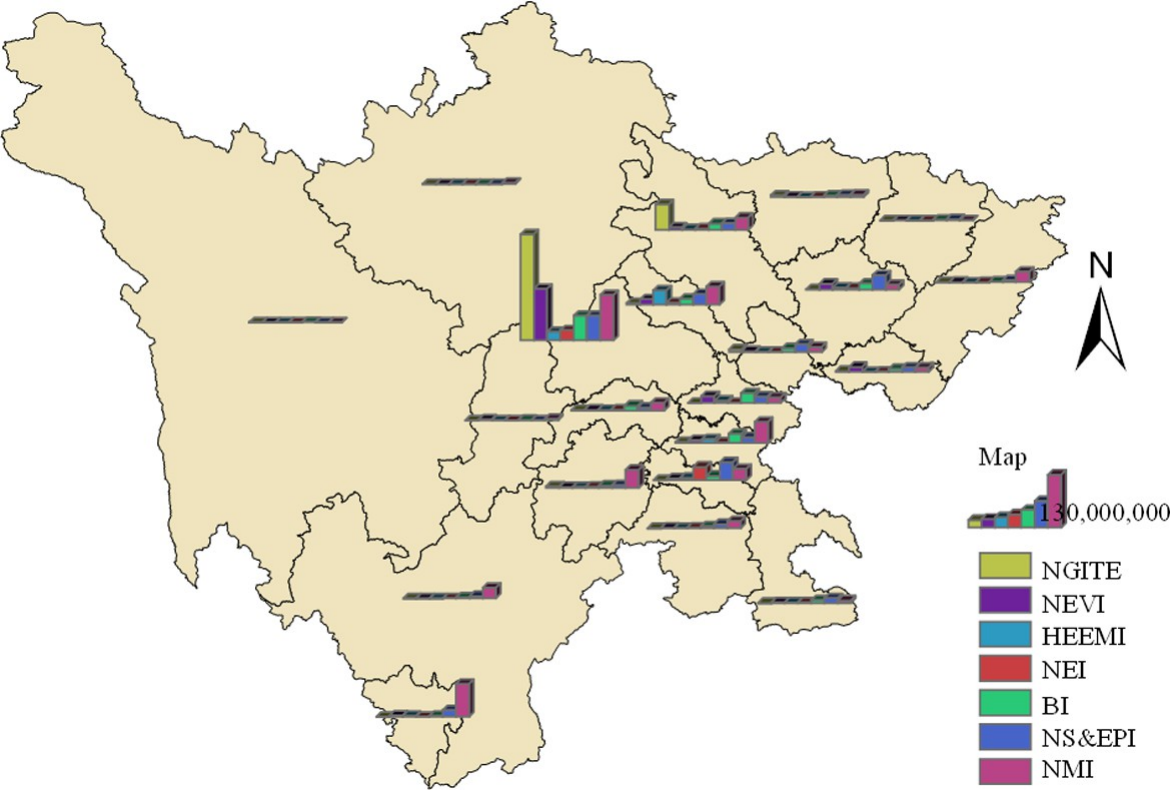
Distribution of business income by industry of sample points of strategic emerging industries in Sichuan province. The bottom map data from the website: http://datav.aliyun.com/portal/school/atlas/area_selector. The source data is from Autonavi Open platform.

From the absolute analysis of operating income, the income level of the new generation information technology industry, new energy automobile industry and new material industry is very high. The new generation information technology industry has the best development among all strategic emerging industries, and the industry income level has obvious advantages over other industries. As showed in Fig 8. According to Fig 8, the new generation information technology industry and the new energy vehicle industry have great potential in increasing social wealth, which is another proof that we recommend priority development.

### Spatial distribution characteristics

#### Unbalanced industrial development

**Fig 9:**
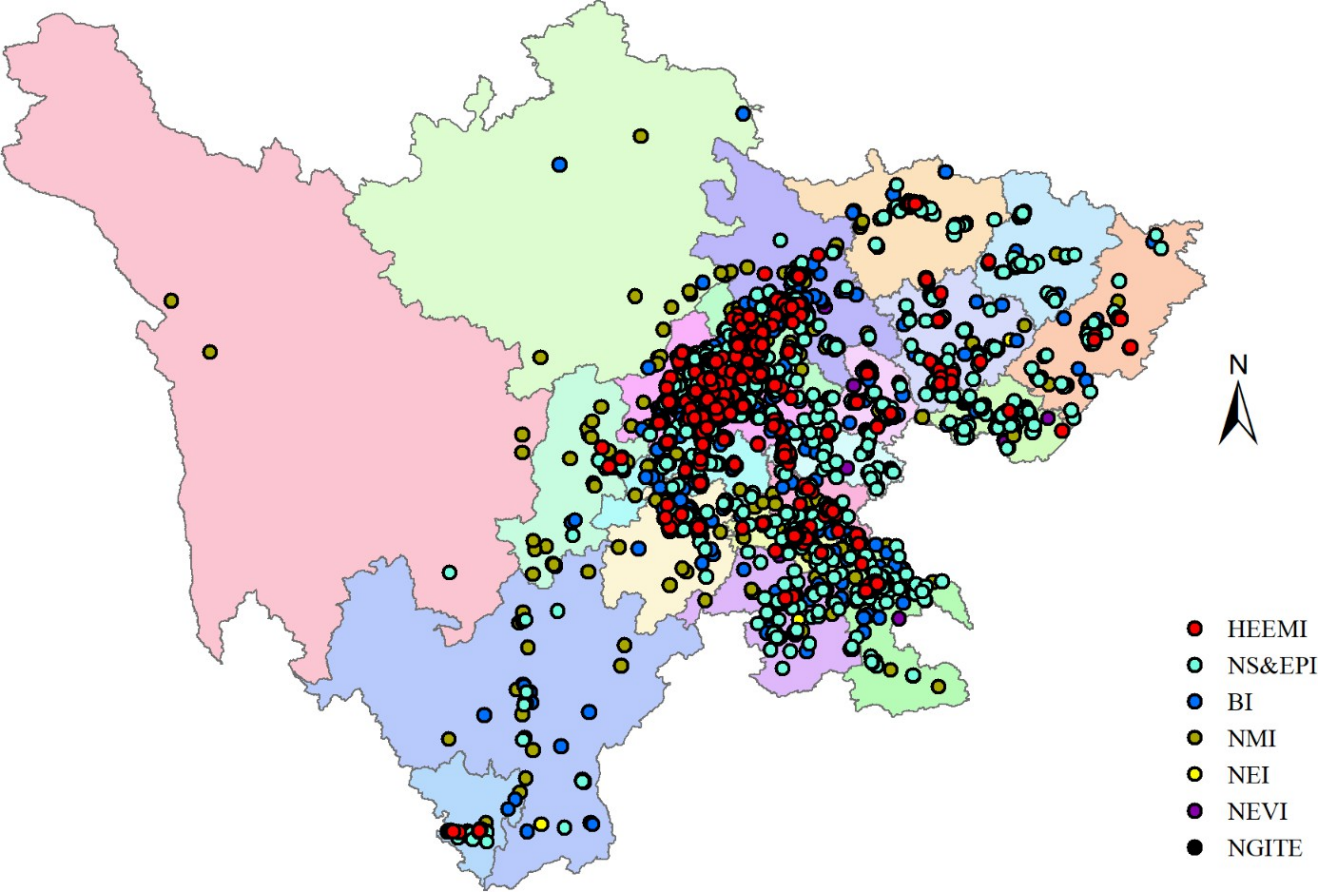
Distribution status of strategic emerging industries in Sichuan province. The bottom map data from the website: http://datav.aliyun.com/portal/school/atlas/area_selector. The source data is from Autonavi Open platform.

According to the seven types of strategic emerging industries defined in the "Decision", the development of strategic emerging industries in Sichuan province is not balanced. Biological industry, energy conservation and environmental protection industry and new material industry have begun to take shape and developed rapidly, forming a certain industrial agglomeration area, these industries are mainly concentrated in Chengdu Plain Economic Zone and South Sichuan Economic zone; On the other hand, the new-generation information technology industry, new energy automobile industry, high-end equipment manufacturing industry and new energy industry are relatively small in scale, and the industrial agglomeration is not particularly obvious. They are mainly distributed in Chengdu, Deyang, Mianyang, and other regions of the Chengdu Plain Economic Zone (As showed in Fig 9).

#### Unbalanced regional development

In Fig 9, Chengdu Plain Economic Zone (Chengdu, Mianyang, Deyang, Suining, Ziyang, Meishan, Ya’an, and Leshan) has obvious industrial agglomeration and complete categories of industrial development. It is the key development area of strategic emerging industries in Sichuan province, relying on Leshan high tech Industrial Development Zone, Suining economic and Technological Development Zone, Mianyang Economic and Technological Development Zone Deyang economic and Technological Development Zone, Deyang high tech Industrial Development Zone, Chengdu high tech Industrial Development Zone and Chengdu Economic and Technological Development Zone. In particularly, Chengdu’s Western Industrial Park focuses on the development of advanced manufacturing industry, highlights the development of new generation of strategic emerging industries focusing on information technology, biology, high-end equipment manufacturing, energy conservation and environmental protection, and builds a high-end industrial base with world influence. Biological industry, energy conservation and environmental protection industry and new material industry are better developed in the four regions included in South Sichuan Economic Zone, while the development of new generation information technology, new energy vehicles, high-end equipment manufacturing and new energy industry is relatively slow. Advantageous industries in Northeast Sichuan economic zone are similarly to those in South Sichuan Economic Zone, but the overall development situation is not as good as that in South Sichuan Economic Zone. Except for three types of industries with good progress, other strategic emerging industries are developing very slowly.

#### Industrial competition and reconstruction

Energy saving and environmental protection industry has obvious development advantages in Chengdu Plain Economic Zone and southern Sichuan Economic Zone. Reconstruction phenomenon refers to the phenomenon of repeated construction due to high similarity of industrial structure. The phenomenon of industrial restructuring in Northeast Sichuan Economic Zone and South Sichuan Economic Zone is relatively serious. Both tend to vigorously develop biology, energy conservation and environmental protection and new material industries, while other types of industries improve slowly. Within the Chengdu Plain Economic Zone, which has absolute advantages in development, Chengdu, Mianyang and Deyang all have strategic emerging industries trying to include all types. This phenomenon is not conducive to the formation of industrial clusters and the overall development of strategic emerging industries in Sichuan province. For example, the energy conservation and environmental protection industry has a great momentum of development in Chengdu Plain, southern Sichuan, and Northeast Sichuan economic zones, which weakens the advantages of industrial agglomeration development to a certain extent, and it is inevitable that there are adverse phenomena such as vicious competition among regions.

## Conclusions

Strategic emerging industries have played an extremely important role in stabilizing growth, adjusting structure, promoting transformation, and benefiting people’s livelihood. Therefore, Sichuan, located in the southwest border of the motherland, should scientifically select strategic emerging industries in line with its own development advantages, find its own positioning, find a good focus, and make effective efforts, which will surely contribute to the revitalization of the West. This paper holds that it is scientific for Sichuan province to choose the new generation of information technology, new energy, high-end equipment manufacturing, new materials, biology, energy conservation and environmental protection industries as the layout of its own strategic emerging industries, but it should also pay attention to making a certain balance among these seven industries to fully develop the most potential strategic emerging industries in line with the actual situation of Sichuan are particularly important, and the phenomenon of full force and convergence with other provinces and cities should be avoided. Taking the layout of strategic emerging industries in Sichuan province as an example, this paper studies the priority of selecting and developing strategic emerging industries in a region. By constructing a reasonable evaluation index system and combining AHP with FCEM, the following conclusions are drawn: Firstly, in the development process of strategic emerging industries in Sichuan province, the priority of industrial development can be scientifically arranged according to the regional advantages. The research results believe that Sichuan province should give priority to the development of new generation information technology industry and new energy vehicle industry, then high-end equipment manufacturing industry, energy-saving and environmental protection industry and new energy industry, and finally biological industry and new material industry. The coefficient of influence and coefficient of sensitivity have different influences on the comprehensive score of strategic emerging industries. The larger the coefficient of influence is, the higher the comprehensive score is, while the larger the coefficient of sensitivity is, the lower the comprehensive score is. Secondly, the number of enterprises in the new-generation information technology industry and the new energy vehicle industry is still not dominant in Sichuan province. Their total industrial output value contributes the most to the Chengdu Plain Economic Zone, and their contribution to employment is not outstanding, but they show great benefits in increasing social wealth. Finally, the study found that the current development of strategic emerging industries in Sichuan province is extremely unbalanced in various regions, and the phenomenon of competition and reconstruction is obvious, which hinders the development and growth of strategic emerging industries to a certain extent. The research results not only provide strong support for the development decision of strategic emerging industries in Sichuan province, but also provide guidance for other regions in the world in the development of strategic emerging industries.

This paper studies the priority of the overall strategic emerging industrial development in Sichuan province, which has a certain value. The deployment of strategic emerging industries from the Party Central Committee to provinces, cities, districts and counties to the enterprise level, each level will affect the development of the industry. Each level is of great importance and indispensable. Therefore, in the future, we can study the strategic emerging industry selection of cities, districts and counties in Sichuan province, or the location selection of strategic emerging industries and the micro location of enterprises, which is believed to play a guiding role in the development of strategic emerging industries in Sichuan province. When the updated data can be used, we can also use the latest available data for further tracking and analysis to grasp the dynamic changes in the development of strategic emerging industries. The current research focuses on the layout and development of strategic emerging industries in Sichuan province. In the future, we can carry out similar analysis and research from the regional and national levels.

This study also has limitations. Firstly, this paper does not use other MCDM method (such as BWM, COPRAS, etc.) or non-fuzzy methods for comparative research. Secondly, the evaluation index system constructed in this paper adopts AHP and FCEM to assign weight and score. However, these two methods depend on expert judgment to a certain extent and have certain subjectivity, which may lead to deviation of the results. Finally, due to the limitations of available data, the research scope of this paper is limited to the strategic emerging industries in Sichuan province and its subordinate areas during the 12th Five-Year Plan period.

## Supporting information

S1 Appendix Calculation of influence coefficient and sensitivity coefficient of relevant industries in Sichuan province. (DOCX)

S2 Appendix R matrix of strategic emerging industries. (DOCX)

S3 Appendix Relevant data. (XLSX)

## References

[1] Yue H. Research on spatial distribution of strategic emerging industries: Capital University of Economics and Business; 2014.

[2] LiChao N. Research on the development and evolution of strategic emerging industries: Capital University of Economics and Business; 2011.

[3] Tu Wenming, Dunhu L. The paradigm evolution and realization mechanism of regional agglomeration of strategic emerging industries. Scientific and technological progress and countermeasures. 2015;32(02):73–8.

[4] Huang C, Cai Y, Chen C. Situation and Countermeasure of Free Ports and Marine Strategic Emerging Industries: A Case of Zhoushan in China. Journal of Coastal Research. 2020:252–7. doi: 10.2112/si103-054.1 PubMed PMID: WOS:000543720600054.

[5] Guo X, Hui X. Research on Regional Strategic Emerging Industry Selection Models Based on Fuzzy Optimization and Entropy Evaluation. Journal of Applied Mathematics. 2012;2012:1–15. doi: 10.1155/2012/120393 PubMed PMID: WOS:000312805400001.

[6] Cao LJ. An application of SWOT analysis in the research of strategic emerging industry talents in Henan province of China. Basic & Clinical Pharmacology & Toxicology. 2020;126:16-. PubMed PMID: WOS:000515558400029.31468698

[7] Jian LR, Wang DF, Wang DA. Evolution Mechanism of Strategic Emerging Industrial Clusters Based on Hybridization of Grey Number and Optimized Scale-Free Network. Journal of Grey System. 2021;33(1):138–55. PubMed PMID: WOS:000663588700009.

[8] Hong-qi wang, Sue hongyan, Jianlong W. Research on spatial distribution method of strategic emerging industries and its application. China Science and Technology Forum. 2013;04:28–34.

[9] Sun Y, Lu Y, Wang T, Ma H, He G. Pattern of patent-based environmental technology innovation in China. Technological Forecasting and Social Change. 2008;75(7):1032–42. doi: 10.1016/j.techfore.2007.09.004

[10] Massard N, Mehier C. Proximity and Innovation through an ‘Accessibility to Knowledge’ Lens. Regional Studies. 2009;43(1):77–88. doi: 10.1080/00343400701808881

[11] Mukim M. Does Agglomeration Boost Innovation? An Econometric Evaluation. Spatial Economic Analysis. 2012;7(3):357–80. doi: 10.1080/17421772.2012.694142

[12] Bradford N, Wolfe DA. Governing regional economic development: innovation challenges and policy learning in Canada. Cambridge Journal of Regions, Economy and Society. 2013;6(2):331–47. doi: 10.1093/cjres/rst006

[13] Park J-I, Lee S. Examining the spatial patterns of green industries and the role of government policies in South Korea: Application of a panel regression model (2006–2012). Renewable and Sustainable Energy Reviews. 2017;78:614–23. doi: 10.1016/j.rser.2017.04.061

[14] Fahmi FZ, Koster S, van Dijk J. The location of creative industries in a developing country: The case of Indonesia. Cities. 2016;59:66–79. doi: 10.1016/j.cities.2016.06.005

[15] Chen X, Wang Y. Research on financing efficiency of China’s strategic emerging industries based on super efficiency DEA and tobit model. International Journal of Emerging Markets. 2020; ahead-of-print(ahead-of-print). doi: 10.1108/ijoem-02-2020-0188 PubMed PMID: WOS:000576410900001.

[16] Sun H, Geng C. ENVIRONMENTAL RESEARCH ON FINANCING EFFICIENCY AND DYNAMIC ADJUSTMENT OF CAPITAL STRUCTURE OF STRATEGIC EMERGING INDUSTRIES. Journal of Environmental Protection and Ecology. 2019;20(3):1586–97. PubMed PMID: WOS:000497992700056.

[17] Wang Q, Geng C-x. Research on Financing Efficiencies of Strategic Emerging Listed Companies by Six-Stage DEA Model. Mathematical Problems in Engineering. 2017;2017:1–8. doi: 10.1155/2017/3284657 PubMed PMID: WOS:000405343600001.

[18] Luo Q, Miao C, Sun L, Meng X, Duan M. Efficiency evaluation of green technology innovation of China’s strategic emerging industries: An empirical analysis based on Malmquist-data envelopment analysis index. Journal of Cleaner Production. 2019;238. doi: 10.1016/j.jclepro.2019.117782 PubMed PMID: WOS:000487231200045.

[19] Miao C, Fang D, Sun L, Luo Q, Yu Q. Driving effect of technology innovation on energy utilization efficiency in strategic emerging industries. Journal of Cleaner Production. 2018;170:1177–84. doi: 10.1016/j.jclepro.2017.09.225 PubMed PMID: WOS:000414879300104.

[20] Zeng G, Guo H, Geng C. A Five-Stage DEA Model for Technological Innovation Efficiency of China’s Strategic Emerging Industries, Considering Environmental Factors and Statistical Errors. Polish Journal of Environmental Studies. 2021;30(1):927–41. doi: 10.15244/pjoes/123287 PubMed PMID: WOS:000592544100004.

[21] Zhong Z, Meng F, Zhu Y, Wang G. Research on the Technological Innovation Efficiency of China’s Strategic Emerging Industries Based on SBM: NDEA Model and Big Data. Mathematical Problems in Engineering. 2020;2020:1–11. doi: 10.1155/2020/7069191 PubMed PMID: WOS:000537266700007.

[22] Yanwei Lv, Hui S. Agglomeration degree evolution and spatial layout of China’s strategic emerging industries. Regional research and development. 2013;32(04):15–21.

[23] Ting L. Agglomeration of strategic emerging industries and influencing factors: Zhejiang Gongshang University; 2013.

[24] Zengjia L. Spatial statistical analysis of regional circulation industry development differences: Zhejiang Gongshang University; 2013.

[25] Yu-ming wu, he J-k. Spatial econometric analysis of R&D spillover and regional innovation cluster. Journal of Management Science. 2008;11(04):59–66.

[26] Zadeh LA. Fuzzy sets. Information and Control. 1965;8(3):338–53.

[27] Lu Z, Shen Y. The study on venture capital project appraisal using AHP-fuzzy comprehensive evaluation methods. International Journal of Advancements in Computing Technology. 2011;3(8):50–6.

[28] Lin J, Li T., Zhao Z, Zheng W., Liu T. Assessment on power system black-start schemes based on entropy-weighted fuzzy comprehensive evaluation model. Power System Technology. 2012;36(2):115–20.

[29] Chengcheng B, Junhai M, editors. The application of fuzzy comprehensive evaluation model in the performance assessment of state-owned enterprises. Proceedings of 3rd International Symposium on Computational Intelligence and Design, IEEE Computer Society; 2010; Hangzhou.

[30] Afful-Dadzie A, Afful-Dadzie E, Nabareseh S, Oplatkova ZK. Tracking progress of African Peer Review Mechanism (APRM) using fuzzy comprehensive evaluation method. Kybernetes. 2014;43(8):1193–208. doi: 10.1108/k-03-2014-0049 PubMed PMID: WOS:000343399700007.

[31] Rezaei J. Best-worst multi-criteria decision-making method. Omega-International Journal of Management Science. 2015;53:49–57. doi: 10.1016/j.omega.2014.11.009 PubMed PMID: WOS:000350936000006.

[32] Rezaei J. Best-worst multi-criteria decision-making method: Some properties and a linear model. Omega-International Journal of Management Science. 2016;64:126–30. doi: 10.1016/j.omega.2015.12.001 PubMed PMID: WOS:000381535000010.

[33] Zavadskas EK, Kaklauskas A, Sarka V. The new method of multicriteria complex proportional assessment of projects. Technological and Economic Development of Economy. 1994;1(3):131–9.

[34] Podvezko V. The Comparative Analysis of MCDA Methods SAW and COPRAS. Inzinerine Ekonomika-Engineering Economics. 2011;22(2):134–46. PubMed PMID: WOS:000290236200002.

[35] Kaklauskas A, Zavadskas EK, Raslanas S, Ginevicius R, Komka A, Malinauskas P. Selection of low-e windows in retrofit of public buildings by applying multiple criteria method COPRAS: A Lithuanian case. Energy and Buildings. 2006;38(5):454–62. doi: 10.1016/j.enbuild.2005.08.005 PubMed PMID: WOS:000236653400008.

[36] Chakraborty S, Zavadskas E, Antucheviciene J. Applications of waspas method as a multi-criteria decision-making tool. Economic computation and economic cybernetics studies and research / Academy of Economic Studies. 2015;49:5–22.

[37] Zavadskas EK, Turskis Z, Antucheviciene J, Zakarevicius A. Optimization of Weighted Aggregated Sum Product Assessment. Elektronika Ir Elektrotechnika. 2012;122(6):3–6. doi: 10.5755/j01.eee.122.6.1810 PubMed PMID: WOS:000305717900001.

[38] Keshavarz-Ghorabaee M, Amiri M, Zavadskas EK, Turskis Z, Antucheviciene J. Simultaneous Evaluation of Criteria and Alternatives (SECA) for Multi-Criteria Decision-Making. Informatica. 2018;29(2):265–80. doi: 10.15388/Informatica.2018.167 PubMed PMID: WOS:000441412300005.

[39] Keshavarz-Ghorabaee M, Amiri M, Zavadskas EK, Turskis Z, Antucheviciene J. Determination of Objective Weights Using a New Method Based on the Removal Effects of Criteria (MEREC). Symmetry-Basel. 2021;13(4). doi: 10.3390/sym13040525 PubMed PMID: WOS:000643628700001.

[40] Keshavarz Ghorabaee M, Zavadskas EK, Olfat L, Turskis Z. Multi-Criteria Inventory Classification Using a New Method of Evaluation Based on Distance from Average Solution (EDAS). Informatica. 2015;26(3):435–51. doi: 10.15388/Informatica.2015.57

[41] Hwang CL, Yoon KP. Multiple Attribute Decision Making Methods and Applications: A State-of-the Art Survey. London: Springer; 1981.

[42] Opricovic S, Tzeng G-H. Compromise solution by MCDM methods: A comparative analysis of VIKOR and TOPSIS. European Journal of Operational Research. 2004;156(2):445–55. 10.1016/S0377-2217(03)00020-1

[43] Saaty T. The Analytic Hierarchy Process: Planning, Priority Setting, Resource Allocation: McGraw-Hill. New York. 1980.

[44] Cui L. Applying Fuzzy Comprehensive Evaluation Method to Evaluate Quality in Crisis and Emergency Management. Communications in Statistics—Theory and Methods. 2012;41(21):3942–59. doi: 10.1080/03610926.2012.691197

[45] Wang Y, Yang W, Li M, Liu X. Risk assessment of floor water inrush in coal mines based on secondary fuzzy comprehensive evaluation. International Journal of Rock Mechanics and Mining Sciences. 2012;52:50–5. 10.1016/j.ijrmms.2012.03.006.

[46] Zhang M, Yang W. Fuzzy comprehensive evaluation method applied in the real estate investment risks research. Physics Procedia. 2012;24:1815–21.

[47] Feng L, Zhu XD, Sun X. Assessing coastal reclamation suitability based on a fuzzy-AHP comprehensive evaluation framework: A case study of Lianyungang, China. Marine Pollution Bulletin. 2014;89(1–2):102–11. doi: 10.1016/j.marpolbul.2014.10.029 PubMed PMID: WOS:000347494700028. 25455377

[48] He DY, Zhang QJ. The application of analytic hierarchy process and fuzzy comprehensive evaluation method for the evaluation of enterprise training effectiveness. International Journal of Computational Science and Engineering. 2017;14(2):126–34. doi: 10.1504/ijcse.2017.10003826 PubMed PMID: WOS:000405548900003.

[49] Raihan AS, Ali SM, Roy S, Das M, Kabir G, Paul SK. Integrated Model for Soft Drink Industry Supply Chain Risk Assessment: Implications for Sustainability in Emerging Economies. International Journal of Fuzzy Systems. 2021. doi: 10.1007/s40815-020-01039-w PubMed PMID: WOS:000637646100001.

[50] Wang X, Li X, Zhen F, Zhang JH. How smart is your tourist attraction?: Measuring tourist preferences of smart tourism attractions via a FCEM-AHP and IPA approach. Tourism Management. 2016;54:309–20. doi: 10.1016/j.tourman.2015.12.003 PubMed PMID: WOS:000372560100031.

[51] Yue H. Spatial distribution of strategic emerging Industries: A case study of Beijing: Capital University of Economics and Business; 2014.

[52] Jiang ning C. Development evaluation and path Selection of China’s strategic emerging industries: Hebei university; 2015.

[53] Krugman P. Geography and Trade MIT Press, Cambridge, MA.; 1991.

[54] Lucheng Huang, Xiaomei Luo, Hong Miao. Evaluation index and standard of development effect of strategic emerging industries. Scientific and technological progress and Countermeasures. 2012;29(24):136–9.

[55] Saaty TL, Peniwati K. Group Decision Making: Drawing out and Reconciling Differences: Pittsburgh, Pennsylvania: RWS Publications.; 2008.

