# Supplementary material for: Strategic emerging industry layout based on analytic hierarchy process and fuzzy comprehensive evaluation: A case study of Sichuan province
Source: PLoS One. 2022 Mar 4;17(3):e0264578. doi: 10.1371/journal.pone.0264578 (PMC8896735; doi:10.1371/journal.pone.0264578)
Supplement: S1 Appendix — (DOCX) [file pone.0264578.s001.docx]

| Input-output table（2012） | Coefficient of influence | Coefficient of sensitivity |
| --- | --- | --- |
| Agriculture, forestry, animal husbandry  and fishery  Coal mining and washing industry  Oil and gas extraction industry  Metal ore mining and dressing industry  Mining and beneficiation of non-metallic and other minerals  Food manufacturing and tobacco processing industry  textile industry  Textile, clothing, shoes, hats, leather, down and its products industry  Wood processing and furniture manufacturing  Paper printing and cultural, educational, and sporting goods manufacturing  Petroleum processing, coking and nuclear fuel processing industry  chemical industry  Non-metallic mineral products industry  Metal smelting and calendering industry  Metal manufacturing  General and special equipment manufacturing  Transportation equipment manufacturing  Electrical machinery and equipment manufacturing  Manufacturing of communication equipment, computers, and other electronic equipment  Instrument and cultural office machinery manufacturing  Arts and crafts and other manufacturing industries  Scrap  Production and supply of electricity and heat  Gas production and supply industry  Water production and supply  construction  Transportation and storage industry  Postal industry  Information transmission, computer services and software industry  Wholesale and retail  Accommodation and catering  finance  real estate  Leasing and business services  Research and experimental development industry  Comprehensive technical service industry  Water conservancy, environment, and public facilities management  Residential services and other services  education  Health, social security, and social welfare  Culture, sports, and entertainment  Public administration and social organizations | 0.7008  1.0247  0.9223  0.9955  1.0338  0.9243  1.0995  1.1374  1.1463  1.0718  1.1097  1.0990  1.1103  1.1910  1.1987  1.2223  1.2281  1.2436  1.2368  1.2565  1.1974  1.1166  0.4928  1.2647  1.0464  1.0467  0.9356  1.1474  0.6916  0.8865  0.8584  0.8506  0.6249  0.6248  1.0360  0.8312  0.9135  0.8960  0.7424  0.9077  0.9162  0.7379 | 1.2371  1.1570  1.0097  0.7640  0.7419  0.9656  0.6930  0.4759  0.5561  0.8565  0.9093  2.4665  0.6338  2.3411  0.6415  0.8473  0.5597  0.7457  0.7417  1.1875  0.3701  0.3233  0.3921  0.4058  1.5882  0.3223  0.3474  0.3430  0.9022  0.7345  0.7288  0.5445  1.1202  0.4093  0.5087  0.3670  0.3123  0.4728  0.2958  0.2806  0.3237  0.2826 |
