# Supplementary material for: Strategic emerging industry layout based on analytic hierarchy process and fuzzy comprehensive evaluation: A case study of Sichuan province
Source: PLoS One. 2022 Mar 4;17(3):e0264578. doi: 10.1371/journal.pone.0264578 (PMC8896735; doi:10.1371/journal.pone.0264578)
Supplement: S2 Appendix — (DOCX) [file pone.0264578.s002.docx]

|  |  | Not suitable | Suitable | More suitable | Very suitable |
| --- | --- | --- | --- | --- | --- |
| NS&EPI |  |  |  |  |  |
| $u_{11}$ |  | 0.1654 | 0.8346 | 0.0000 | 0.0000 |
| $u_{12}$ |  | 0.0000 | 0.8720 | 0.1280 | 0.0000 |
| $u_{13}$ |  | 0.0000 | 0.2910 | 0.7090 | 0.0000 |
| $u_{21}$ |  | 0.0000 | 0.1510 | 0.8490 | 0.0000 |
| $u_{22}$ |  | 0.5698 | 0.4302 | 0.0000 | 0.0000 |
| $u_{31}$ |  | 0.0000 | 0.5278 | 0.4722 | 0.0000 |
| $u_{32}$ |  | 0.0000 | 0.0000 | 0.0500 | 0.9500 |
| $u_{41}$ |  | 0.0000 | 0.6750 | 0.3250 | 0.0000 |
| $u_{42}$ |  | 0.0000 | 0.1164 | 0.8836 | 0.0000 |
| $u_{43}$ |  | 0.0000 | 0.6639 | 0.3361 | 0.0000 |
| $u_{51}$ |  | 0.2000 | 0.8000 | 0.0000 | 0.0000 |
| $u_{52}$ |  | 0.5633 | 0.4367 | 0.0000 | 0.0000 |
| $u_{53}$ |  | 0.0000 | 0.4613 | 0.5387 | 0.0000 |
| BI |  |  |  |  |  |
| $u_{11}$ |  | 0.6385 | 0.3615 | 0.0000 | 0.0000 |
| $u_{12}$ |  | 0.0000 | 0.9810 | 0.0190 | 0.0000 |
| $u_{13}$ |  | 0.0000 | 0.9300 | 0.0700 | 0.0000 |
| $u_{21}$ |  | 0.0000 | 0.0000 | 0.8495 | 0.1505 |
| $u_{22}$ |  | 0.0000 | 0.8882 | 0.1118 | 0.0000 |
| $u_{31}$ |  | 0.1338 | 0.8662 | 0.0000 | 0.0000 |
| $u_{32}$ |  | 0.0000 | 0.7000 | 0.3000 | 0.0000 |
| $u_{41}$ |  | 0.0000 | 0.8440 | 0.1560 | 0.0000 |
| $u_{42}$ |  | 0.0000 | 0.6856 | 0.3144 | 0.0000 |
| $u_{43}$ |  | 0.0000 | 0.5337 | 0.4663 | 0.0000 |
| $u_{51}$ |  | 0.3600 | 0.6400 | 0.0000 | 0.0000 |
| $u_{52}$ |  | 0.3700 | 0.6300 | 0.0000 | 0.0000 |
| $u_{53}$ |  | 0.0000 | 0.1871 | 0.8129 | 0.0000 |
| NEI |  |  |  |  |  |
| $u_{11}$ |  | 0.0000 | 0.8800 | 0.1200 | 0.0000 |
| $u_{12}$ |  | 0.0000 | 0.9580 | 0.0420 | 0.0000 |
| $u_{13}$ |  | 0.0000 | 0.0000 | 0.5040 | 0.4960 |
| $u_{21}$ |  | 0.0000 | 0.0000 | 0.5815 | 0.4185 |
| $u_{22}$ |  | 0.0000 | 0.8241 | 0.1759 | 0.0000 |
| $u_{31}$ |  | 0.1158 | 0.8842 | 0.0000 | 0.0000 |
| $u_{32}$ |  | 0.1667 | 0.8333 | 0.0000 | 0.0000 |
| $u_{41}$ |  | 0.6220 | 0.3780 | 0.0000 | 0.0000 |
| $u_{42}$ |  | 0.0000 | 0.7048 | 0.2952 | 0.0000 |
| $u_{43}$ |  | 0.1170 | 0.8830 | 0.0000 | 0.0000 |
| $u_{51}$ |  | 0.7000 | 0.3000 | 0.0000 | 0.0000 |
| $u_{52}$ |  | 0.4767 | 0.5233 | 0.0000 | 0.0000 |
| $u_{53}$ |  | 0.0000 | 0.0000 | 0.0000 | 1.0000 |
| HEEMI |  |  |  |  |  |
| $u_{11}$ |  | 0.7646 | 0.2354 | 0.0000 | 0.0000 |
| $u_{12}$ |  | 0.0000 | 0.0000 | 0.3420 | 0.6580 |
| $u_{13}$ |  | 0.0197 | 0.9803 | 0.0000 | 0.0000 |
| $u_{21}$ |  | 0.0000 | 0.0000 | 0.7860 | 0.2140 |
| $u_{22}$ |  | 0.0000 | 0.8146 | 0.1854 | 0.0000 |
| $u_{31}$ |  | 0.7168 | 0.2832 | 0.0000 | 0.0000 |
| $u_{32}$ |  | 0.6667 | 0.3333 | 0.0000 | 0.0000 |
| $u_{41}$ |  | 0.0000 | 0.7890 | 0.2110 | 0.0000 |
| $u_{42}$ |  | 0.0000 | 0.0000 | 0.0000 | 1.0000 |
| $u_{43}$ |  | 0.0000 | 0.9356 | 0.0644 | 0.0000 |
| $u_{51}$ |  | 0.0000 | 0.8400 | 0.1600 | 0.0000 |
| $u_{52}$ |  | 0.5333 | 0.4667 | 0.0000 | 0.0000 |
| $u_{53}$ |  | 0.0000 | 0.6314 | 0.3686 | 0.0000 |
| NGITE |  |  |  |  |  |
| $u_{11}$ |  | 0.0338 | 0.9662 | 0.0000 | 0.0000 |
| $u_{12}$ |  | 0.0000 | 0.0000 | 0.4147 | 0.5853 |
| $u_{13}$ |  | 0.0000 | 0.2120 | 0.7880 | 0.0000 |
| $u_{21}$ |  | 0.0000 | 0.0000 | 0.3715 | 0.6285 |
| $u_{22}$ |  | 0.8678 | 0.1322 | 0.0000 | 0.0000 |
| $u_{31}$ |  | 0.0000 | 0.9483 | 0.0517 | 0.0000 |
| $u_{32}$ |  | 0.0000 | 0.7000 | 0.3000 | 0.0000 |
| $u_{41}$ |  | 0.2420 | 0.7580 | 0.0000 | 0.0000 |
| $u_{42}$ |  | 0.0000 | 0.0000 | 0.0000 | 1.0000 |
| $u_{43}$ |  | 0.0000 | 0.0816 | 0.9184 | 0.0000 |
| $u_{51}$ |  | 0.0000 | 0.7700 | 0.2300 | 0.0000 |
| $u_{52}$ |  | 0.0000 | 0.8440 | 0.1560 | 0.0000 |
| $u_{53}$ |  | 0.0000 | 0.5733 | 0.4267 | 0.0000 |
| NMI |  |  |  |  |  |
| $u_{11}$ |  | 0.7654 | 0.2346 | 0.0000 | 0.0000 |
| $u_{12}$ |  | 0.0000 | 0.9910 | 0.0090 | 0.0000 |
| $u_{13}$ |  | 0.0000 | 0.7390 | 0.2610 | 0.0000 |
| $u_{21}$ |  | 0.0000 | 0.0000 | 0.6735 | 0.3265 |
| $u_{22}$ |  | 0.7288 | 0.2712 | 0.0000 | 0.0000 |
| $u_{31}$ |  | 0.0000 | 0.5070 | 0.4930 | 0.0000 |
| $u_{32}$ |  | 0.0000 | 0.4000 | 0.6000 | 0.0000 |
| $u_{41}$ |  | 0.2920 | 0.7080 | 0.0000 | 0.0000 |
| $u_{42}$ |  | 0.0000 | 0.1646 | 0.8354 | 0.0000 |
| $u_{43}$ |  | 0.0000 | 0.0000 | 0.9025 | 0.0975 |
| $u_{51}$ |  | 0.5600 | 0.4400 | 0.0000 | 0.0000 |
| $u_{52}$ |  | 0.5467 | 0.4533 | 0.0000 | 0.0000 |
| $u_{53}$ |  | 0.7572 | 0.2428 | 0.0000 | 0.0000 |
| NEVI |  |  |  |  |  |
| $u_{11}$ |  | 0.1669 | 0.8331 | 0.0000 | 0.0000 |
| $u_{12}$ |  | 0.0000 | 0.9010 | 0.0990 | 0.0000 |
| $u_{13}$ |  | 0.0000 | 0.6400 | 0.3600 | 0.0000 |
| $u_{21}$ |  | 0.0000 | 0.0000 | 0.3430 | 0.6570 |
| $u_{22}$ |  | 0.7063 | 0.2937 | 0.0000 | 0.0000 |
| $u_{31}$ |  | 0.0000 | 0.3615 | 0.6385 | 0.0000 |
| $u_{32}$ |  | 0.8333 | 0.1667 | 0.0000 | 0.0000 |
| $u_{41}$ |  | 0.0000 | 0.4280 | 0.5720 | 0.0000 |
| $u_{42}$ |  | 0.0000 | 0.0000 | 0.0000 | 1.0000 |
| $u_{43}$ |  | 0.0286 | 0.9714 | 0.0000 | 0.0000 |
| $u_{51}$ |  | 0.0000 | 0.7900 | 0.2100 | 0.0000 |
| $u_{52}$ |  | 0.5067 | 0.4933 | 0.0000 | 0.0000 |
| $u_{53}$ |  | 0.0000 | 0.1429 | 0.8571 | 0.0000 |
